# From rules to foundation models: a comprehensive review of machine learning approaches for siRNA design

**DOI:** 10.1093/nargab/lqag099

**Published:** 2026-08-31

**Authors:** Zahra Khodagholi, Niloofar Yousefi

**Affiliations:** Department of Industrial Engineering, University of Central Florida ,4000 Central Florida Blvd.,Orlando, FL 32816,United States; Department of Industrial Engineering, University of Central Florida ,4000 Central Florida Blvd.,Orlando, FL 32816,United States

## Abstract

Small interfering RNAs (siRNAs) are a clinically validated therapeutic modality with eight FDA-approved drugs, yet designing effective siRNAs remains computationally challenging due to complex dependencies on sequence composition, thermodynamic properties, target-site accessibility, and off-target interactions. Over two decades, computational approaches have evolved from empirical heuristics to deep learning systems integrating physical priors with learned representations. We review the complete landscape of machine learning methods for siRNA design, spanning classical scoring rules, pretrained RNA foundation models, transformer-based efficacy predictors, graph neural networks encoding siRNA/messenger RNA interaction topology, off-target prediction frameworks, and chemical modification-aware architectures. Across over 40 studies, we identify convergent findings: hybrid models integrating thermodynamic features with learned representations are among the strongest performers, although this evidence rests largely on single-model ablations and does not establish that foundation-model embeddings specifically are required; graph neural networks with leakage-aware data splitting address pervasive benchmark inflation; and off-target prediction has matured through empirical RNA-seq frameworks and structure-based features. We distinguish throughout between chemically unmodified siRNAs, which dominate public benchmarks, and the fully modified siRNAs used therapeutically, whose efficacy data remain scarce and whose prediction is correspondingly harder. We provide a taxonomy of methods, head-to-head performance comparisons, benchmark dataset descriptions, code availability, biology-informed interpretability analysis with formal saliency validation protocols, and concrete recommendations for advancing siRNA design. Critical gaps in uncertainty quantification, active learning, and prospective experimental validation are identified as priorities for clinical translation.

## Graphical Abstract

**Graphical Abstract:**
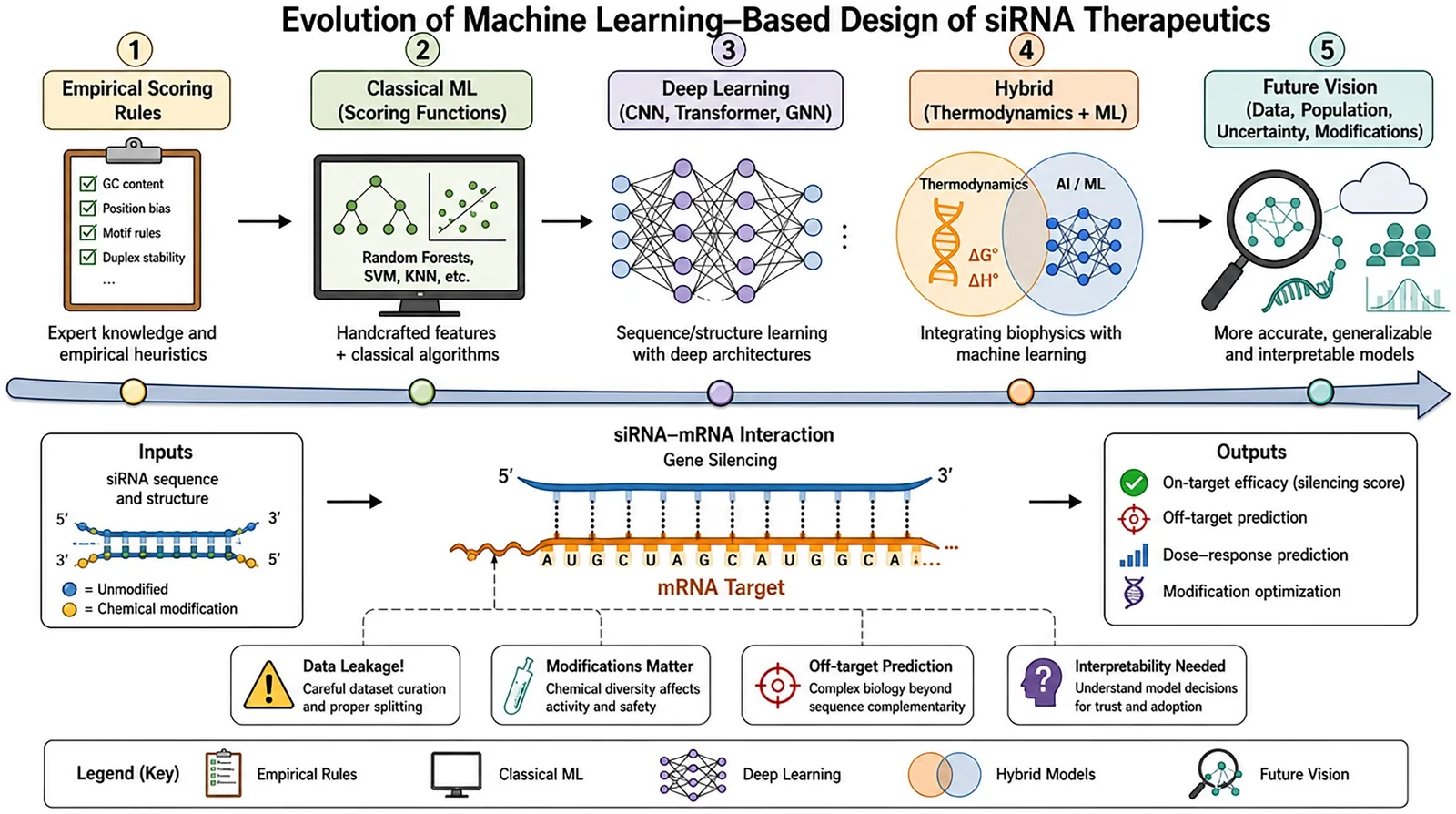

## Introduction

The discovery of RNA interference by [1] in *Caenorhabditis elegans* revealed a conserved biological mechanism by which double-stranded RNA directs sequence-specific gene silencing, recognized with the 2006 Nobel Prize. The subsequent demonstration by [2] that synthetic 21-nucleotide RNA duplexes could trigger potent and specific gene silencing in mammalian cells opened the door to therapeutic exploitation of this pathway. Since then, small interfering RNA (siRNAs) have progressed from laboratory tools to a validated therapeutic class: the FDA has approved patisiran (ONPATTRO, 2018), givosiran (GIVLAARI, 2019), lumasiran (OXLUMO, 2020), inclisiran (LEQVIO, 2021), vutrisiran (AMVUTTRA, 2022), nedosiran (RIVFLOZA, 2023), fitusiran (QFITLIA, 2025), and plozasiran (REDEMPLO, 2025), each targeting a different disease gene through the RNAi pathway [3, 4]. Their clinical success has accelerated computational method development, yet designing effective siRNAs remains a complex computational challenge, because knockdown efficacy depends on a constellation of interacting factors: the thermodynamic profile of the siRNA duplex, the asymmetry between strand ends that governs guide-strand loading into RNA-Induced Silencing Complex (RISC) [5, 6], the accessibility of the target site on the structured messenger RNA (mRNA), seed-mediated off-target interactions that drive unintended gene silencing, and, in therapeutic contexts, the chemical modification patterns that govern nuclease resistance and immunogenicity [7].

The combinatorial space is vast. A 21-nucleotide siRNA drawn from a four-letter alphabet admits ∼$4^{21}$ (4.4 trillion) possible sequences, of which only a tiny fraction will be effective against any given target. Experimental screening alone cannot navigate this space, making computational prediction essential. Over two decades, researchers have developed increasingly sophisticated methods. The earliest approaches distilled experimental observations into positional sequence rules and thermodynamic scoring systems [8–12]. Classical supervised learning, beginning with the BIOPREDsi neural network [13] and extending through support vector machines (SVMs) [14], least absolute shrinkage and selection operator (LASSO) models [15], and multifeature regression approaches [16], demonstrated that machine learning could capture nonlinear feature interactions that individual rules miss. Concurrently, studies of target mRNA secondary structure and accessibility showed that the local structural context around the binding site shapes knockdown efficacy independently of siRNA sequence features [17–19]. The deep learning era brought convolutional architectures for motif detection [20], followed by the transformative impact of pretrained RNA foundation models [21, 22], transformer-based efficacy predictors that fuse thermodynamics with learned embeddings [23–25], graph neural networks that explicitly encode siRNA/mRNA interaction topology [26–28], and biology-informed approaches that encode mechanistic knowledge as differentiable constraints ([29], from the present authors’ group; [30]).

Despite this progress, persistent challenges remain. A recent systematic review by [31] identifies six major obstacles: limited and heterogeneous training data, lack of standardized evaluation protocols, insufficient treatment of chemical modifications, inadequate off-target modeling, poor clinical validation, and limited model interpretability. [26] demonstrate that data leakage between training and test sets has systematically inflated benchmark results across the field, calling into question many previously reported performance comparisons. No formal uncertainty quantification (UQ) methods have been applied to siRNA efficacy prediction despite the safety-critical nature of therapeutic RNA design. And the crucial distinction between knockdown efficacy *prediction* (ranking candidate sequences by expected activity) and *de novo* sequence *design* (generating novel sequences from scratch) remains underappreciated, with the vast majority of published methods addressing the former while sometimes being described as the latter. A related distinction runs throughout this review: the large public benchmarks consist of chemically unmodified siRNAs, whereas therapeutic siRNAs are fully modified, and we separate the two wherever the data allow, since the modified setting is both more clinically relevant and harder to model.

**Figure 1.**
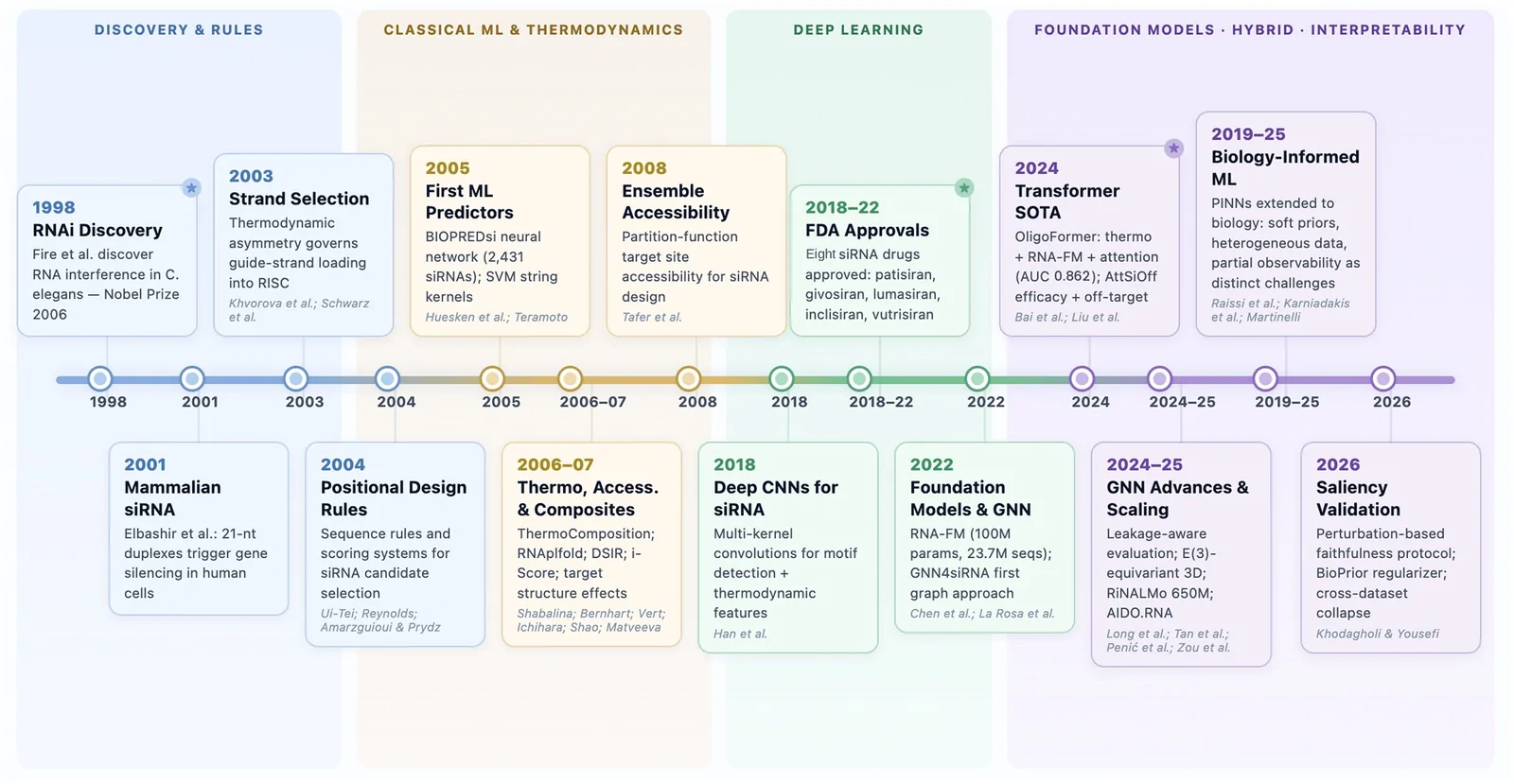
Timeline of computational siRNA design from the discovery of RNA interference **(RNAi)** (1998) to current biology-informed approaches (2026). Upper cards represent paradigm-defining milestones; lower cards show complementary developments within each era. Color bands delineate four methodological eras: discovery and rules, classical Machine Learning (ML) and thermodynamics, deep learning, and foundation models with hybrid interpretability.

This review provides an account of the full methodological landscape, from the biological foundations of RNAi through classical rules and early machine learning to the latest transformer, graph neural networks **(GNNs)**, and foundation model architectures (Fig. 1 traces this evolution from 1998 to 2026). We organize the discussion around method families to help readers understand architectural trade-offs and identify opportunities for contribution. We devote dedicated sections to RNA foundation models (whose ecosystem has expanded rapidly since 2022), biology-informed learning and interpretability (including formal protocols for validating saliency faithfulness), off-target prediction and chemical modification, and benchmark standardization. Throughout, we emphasize the recurring finding that the most effective approaches integrate physical priors with learned representations rather than replacing domain knowledge with end-to-end learning.

### Biological foundations for the computational audience

**Figure 2.**
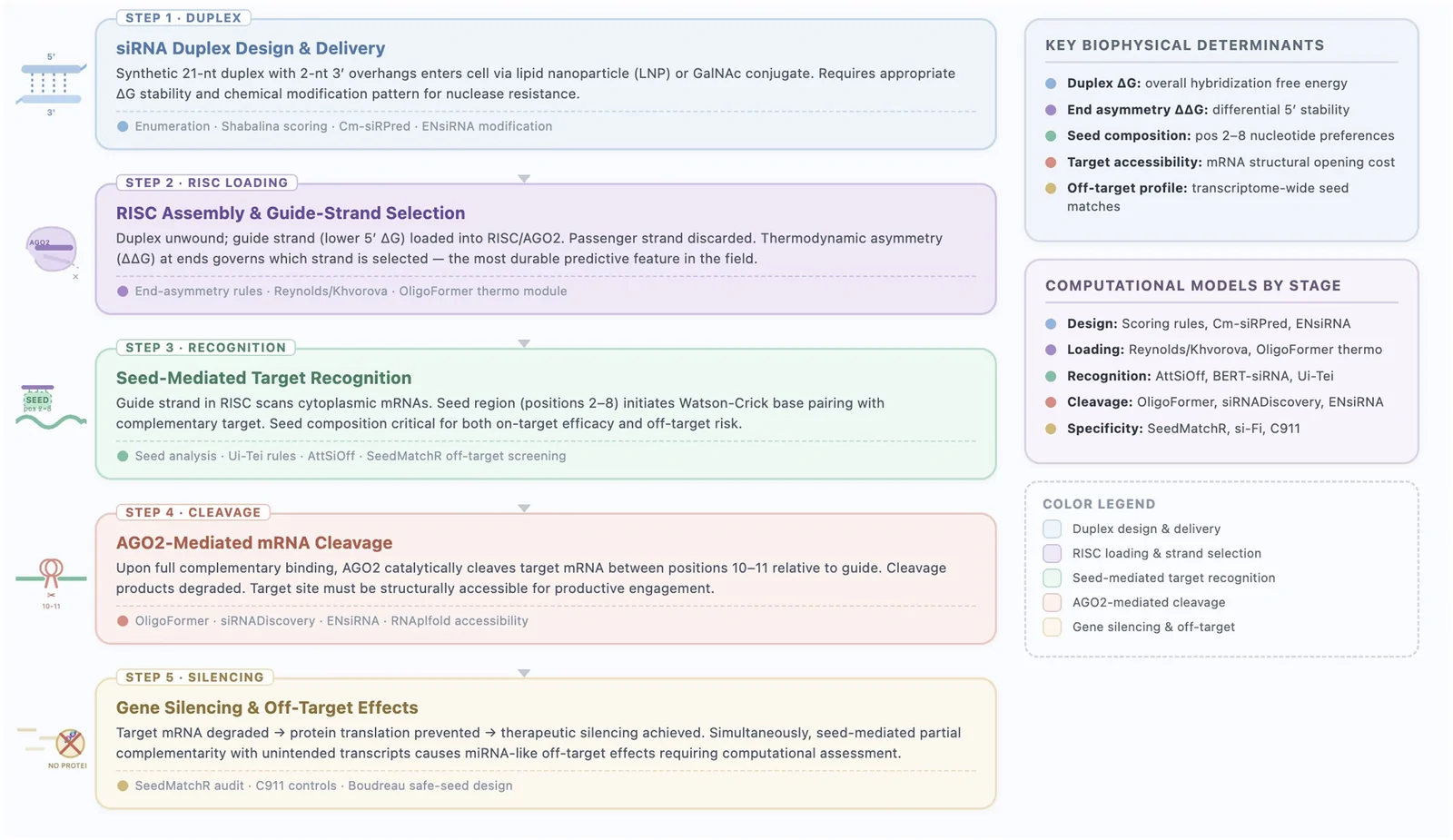
The RNAi pathway and its computational modeling points. Left: five sequential steps from siRNA duplex design through gene silencing, with color-coded stage indicators. Right panels: key biophysical determinants targeted by computational models (top), mapping of specific prediction tools to each pathway stage (middle), and color legend (bottom). Each step presents distinct computational challenges that different architectural families address.

Understanding the RNAi mechanism is essential for appreciating why certain features and architectural choices matter in computational siRNA design (Fig. 2 illustrates the pathway and its computational modeling points). We provide a technically precise account for computational researchers.

#### The RNAi pathway and RISC loading

The RNAi pathway proceeds through well-characterized molecular events, each presenting opportunities for computational optimization. A synthetic siRNA duplex, typically 21 nucleotides in length with 2-nucleotide $3^{\prime }$ overhangs (the structural signature first characterized by [2], enters the cell through delivery vehicles such as lipid nanoparticles or GalNAc conjugates [3]. Once in the cytoplasm, the duplex is recognized by the RNAi machinery. The two strands are unwound and one strand, designated the guide strand, is preferentially loaded into the RISC, whose catalytic core is the Argonaute 2 (AGO2) protein. Strand selection is governed by thermodynamic asymmetry at the duplex termini. [5] and [6], working independently, demonstrated that the strand whose $5^{\prime }$ end has lower thermodynamic stability is preferentially incorporated into RISC, a mechanistic principle that [9] subsequently formalized into quantitative design criteria. This end-asymmetry rule has proven to be among the most durable predictive features in the field, appearing as an explicit input in methods spanning two decades from the earliest scoring algorithms to the latest transformer architectures.

Once loaded, the guide strand within RISC scans cytoplasmic mRNAs for complementary sequences. The seed region, comprising positions 2–8 of the guide strand, initiates target recognition through Watson–Crick base pairing. When full complementarity is established between guide and target, AGO2 catalytically cleaves the mRNA between positions 10 and 11 relative to the guide strand, leading to transcript degradation and gene silencing. The process involves multiple biophysical determinants that computational methods must account for, either through explicit feature engineering or through learned representations.

#### Determinants of knockdown efficacy

Several biophysical factors determine siRNA silencing efficacy, each motivating computational features that recur across all methods reviewed here.

*Thermodynamic properties* encompass the overall free energy of duplex formation (*Δ G*), position-specific stabilities computed across 2-nucleotide sliding windows, and the critical end asymmetry ($\Delta \Delta G_{5^{\prime }}$) that drives strand selection during RISC loading. [11] demonstrated that these calculated thermodynamic features, derived from nearest-neighbor energy parameters, provide predictive value independent of and complementary to sequence composition features. End asymmetry remains among the most robust signals across two decades of method development, consistently improving prediction when added to any feature set.

*Target-site accessibility* refers to the degree to which the mRNA binding site is structurally open and available for RISC engagement. mRNA molecules fold into complex secondary structures, and sites buried within stable hairpins or stem-loops resist productive interaction. [17] provided direct evidence that target secondary structure is a major determinant of knockdown efficacy, independently of siRNA sequence properties. Accessibility can be estimated computationally using partition-function-based methods that consider ensembles of possible structures rather than a single minimum free energy fold. [32] contributed the efficient RNAplfold algorithm for sliding-window computation of local base-pairing probabilities, and [19] and [18] formalized accessibility as a quantitative predictor of silencing efficacy in the contexts of microRNA (miRNA) and siRNA targeting, respectively.

*Sequence composition* captures positional nucleotide preferences that reflect the biochemistry of RISC loading and target cleavage: A/U at the $5^{\prime }$ end of the guide strand, G/C at position 1, A/U richness in the terminal seven positions, and absence of long GC stretches are associated with potent silencing [8, 10]. These rules, while simple, encode genuine mechanistic information about strand-selection thermodynamics and duplex processing.

*Off-target effects* arise primarily through seed-mediated partial complementarity, where the seed region (positions 2–8) of the guide strand binds unintended transcripts in a manner analogous to miRNA-mediated gene regulation. [33] first demonstrated that $3^{\prime }$ UTR seed matches drive off-target transcript silencing, and [34, 35] showed through microarray profiling that this activity is widespread and sequence-dependent. Off-target effects can confound experimental interpretation and pose safety risks in therapeutic contexts, making specificity assessment an essential component of any computational design pipeline.

*Chemical modifications* are required for therapeutic siRNA to achieve adequate nuclease resistance, reduced immunostimulatory activity, and favorable pharmacokinetic properties. Common modifications include $2^{\prime }$-O-methyl and $2^{\prime }$-fluoro substitutions on the ribose sugar, phosphorothioate backbone linkages, and specialized end modifications. [7] reviewed emerging design principles from a drug development perspective, emphasizing that sequence-level choices interact with modification patterns and delivery strategies in ways that early computational methods did not anticipate. These modifications alter duplex geometry and protein interactions, so models trained exclusively on unmodified siRNA data may not transfer reliably to therapeutic contexts [36, 37].

## Classical rules and early machine learning

### Empirical sequence rules (2003–2007)

The computational design of siRNAs began in the early 2000s with systematic experimental studies that distilled effective design principles into reproducible, algorithmic rules. Two landmark studies from 2004 established the foundation upon which all subsequent computational methods have been built. [8] analyzed functional siRNAs across mammalian and chick cell systems and identified four sequence conditions collectively predictive of potent silencing: A/U at position 19 of the sense strand, G/C at position 1, A/U richness in positions 13–19, and absence of GC stretches exceeding 9 nucleotides. These conditions capture basic strand-biasing preferences that reflect the thermodynamics of duplex unwinding and guide-strand selection. Working independently, [9] developed an eight-criteria rational scoring system whose defining contribution was the explicit formalization of thermodynamic asymmetry between the $5^{\prime }$ ends of the two strands as a quantitative predictor of which strand will be loaded into RISC. Their algorithm ranks candidates by the sum of weighted criteria scores, and it became the basis for multiple commercial siRNA design tools that remain in use.

[10] contributed a complementary set of design criteria derived from independent experimental datasets. The partial overlap between the Amarzguioui, Ui-Tei, and Reynolds rules strengthened confidence in the underlying biological principles, while the discrepancies between them highlighted that no single rule set captures all determinants of efficacy, motivating the composite and machine learning approaches that followed. [5] provided crucial mechanistic insight by demonstrating that both siRNAs and endogenous miRNAs exhibit strand bias, with the functionally active strand consistently being the one with lower thermodynamic stability at its $5^{\prime }$ end. This finding, arrived at independently from the [6] demonstration of asymmetric RISC assembly, established end-stability asymmetry as the most important design feature in the entire siRNA prediction literature.

### Thermodynamic models and target accessibility

Building on these sequence rules, [11] took the next step by demonstrating that calculated thermodynamic features, including free energies of duplex formation, terminal dinucleotide stabilities, and nucleotide composition descriptors, add substantial predictive value beyond what positional sequence motifs alone can capture. Their ThermoComposition model showed that physics-informed features and sequence features encode genuinely nonredundant information, establishing the hybrid feature engineering paradigm that remains the dominant approach in the field 20 years later. [16] independently validated the importance of thermodynamic criteria through systematic analysis of experimentally characterized siRNAs, confirming that accessible energy, internal stability profiles, and asymmetry measures improve knockdown prediction when used in combination, and providing a broad comparison of rational design approaches that helped establish best practices for the field.

The role of target mRNA structure in determining knockdown efficacy emerged through several complementary studies. [17] provided direct experimental evidence that target secondary structure is a major and independent determinant of siRNA efficacy, showing that sites embedded in stable mRNA structures resist silencing regardless of the intrinsic quality of the siRNA sequence. This motivated the integration of target accessibility into computational design pipelines. [19] quantified this relationship for miRNA targets, demonstrating that the free energy required to open secondary structure around the binding site strongly predicts silencing efficacy. [18] extended the accessibility framework specifically to siRNA design, showing that ensemble-based accessibility computed from partition-function methods outperforms single-structure predictions. The algorithmic foundation for efficient accessibility computation was provided by [32], whose RNAplfold algorithm enables sliding-window estimation of local base-pairing probabilities in large sequences, making genome-scale accessibility screening computationally tractable.

[12] developed the i-Score Designer, a composite scoring system that integrates multiple existing algorithms through weighted averaging. Their key finding, that optimal weighting varies by target gene characteristics, presaged modern ensemble and multitask learning approaches. [15] contributed an important methodological counterpoint with DSIR, a simple LASSO-based linear model that combines basic sequence and thermodynamic features yet achieves competitive knockdown prediction accuracy. The transparency of this linear model allowed the authors to quantify the contribution of individual features and detect short asymmetric motifs that carry as much predictive information as position-specific nucleotide preferences alone, demonstrating that interpretable models can provide mechanistic insights that complex models obscure.

### The transition to supervised learning

The transition from handcrafted rules to data-driven prediction was marked by BIOPREDsi [13], which trained an artificial neural network on 2431 experimentally characterized siRNAs targeting 34 different mRNAs. This study showed that supervised learning captures nonlinear feature interactions that individual scoring rules cannot represent, and the ‘Huesken dataset’ it produced remains one of the most widely used benchmarks in the field two decades later. [14] explored an alternative machine learning formulation, applying support vector machines with generalized string kernels that operate directly on siRNA sequences without requiring manual feature engineering, demonstrating that kernel-based methods can implicitly capture relevant sequence patterns. Subsequent work explored random forests, gradient-boosted ensembles, and multikernel SVMs, consistently finding that the choice of features matters at least as much as the choice of learning algorithm for knockdown efficacy prediction.

The deep learning era for siRNA arrived with [20], who applied multikernel convolutional neural networks to detect sequence motifs at various scales from the raw nucleotide representation, combined with independently computed thermodynamic features. While convolutional neural networks **(CNNs)** improved upon classical methods for detecting local sequence patterns associated with knockdown efficacy, they inherently struggle with the global context and long-range dependencies that characterize siRNA/mRNA interactions, because convolutional receptive fields capture only local neighborhoods. This limitation motivated the attention-based transformer architectures discussed in ‘Transformer-based architectures for knockdown efficacy prediction’ section, which can model interactions between arbitrary positions in the input.

### Therapeutic context and the prediction-design distinction

**Figure 3.**
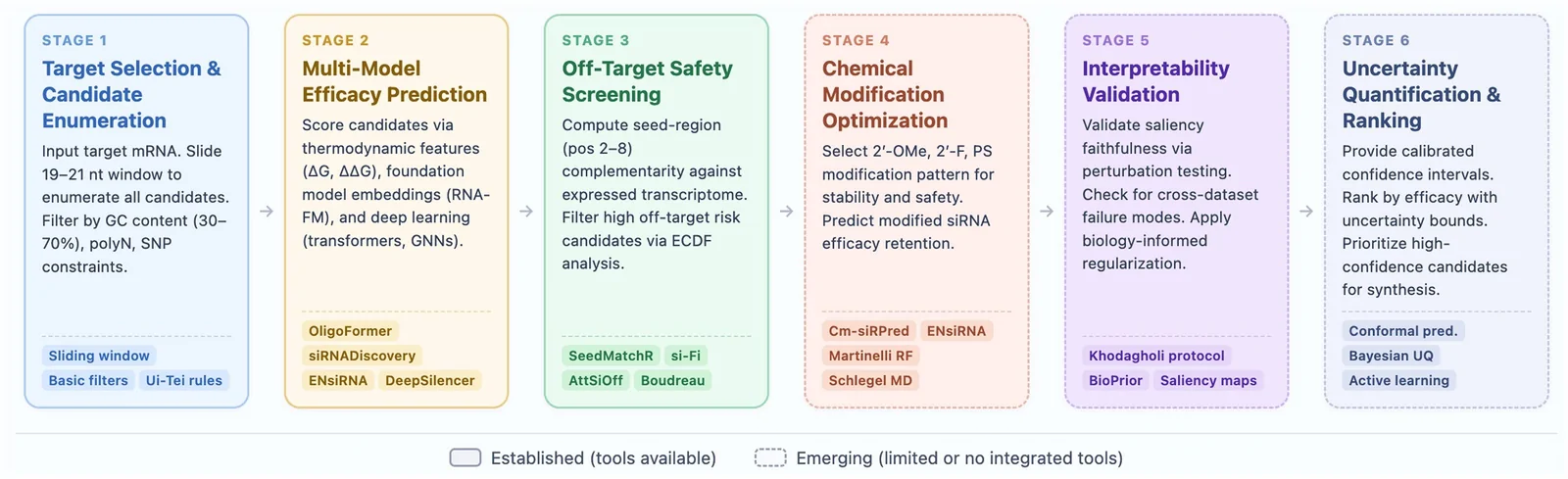
End-to-end computational siRNA design pipeline. Stages 1–3 (solid borders) represent established workflows with available tools. Stages 4–6 (dashed borders) represent emerging or aspirational capabilities with limited or no integrated implementations. Tool labels beneath each stage indicate representative methods reviewed in this paper.

As siRNA progressed from research tools to FDA-approved medicines, researchers recognized that sequence-level prediction cannot be evaluated in isolation from delivery and clinical context. [3] reviewed the full therapeutic landscape of nucleic acid medicines, emphasizing that lipid nanoparticle formulations, GalNAc conjugation strategies, and tissue-targeting approaches fundamentally affect observed potency in ways that sequence-level models do not capture. [7] reviewed emerging design principles from a pharmaceutical development perspective, highlighting interactions between sequence choices, modification patterns, and metabolic stability that create constraints invisible to academic knockdown prediction benchmarks. This context highlights a key distinction: the methods reviewed here primarily address knockdown efficacy *prediction* (ranking candidate sequences by expected potency) rather than *de novo* sequence *design* (generating novel sequences from scratch). The practical workflow involves enumerating all possible candidates along a target mRNA by sliding window, predicting knockdown efficiency for each, and ranking the results, with subsequent filtering for off-target safety, modification compatibility, and developability. Figure 3 illustrates this end-to-end pipeline, distinguishing established stages from emerging capabilities.

## RNA foundation models as pretrained representations

Large pretrained language models for RNA have profoundly reshaped computational siRNA design. Using the self-supervised pretraining paradigm from natural language processing, these foundation models learn general-purpose representations from massive unlabeled corpora, capturing evolutionary conservation, structural propensities, and functional regularities far beyond what siRNA-specific datasets can teach. Since 2022, the RNA foundation model ecosystem has expanded rapidly, with over a dozen models now available spanning a range of architectures, training corpora, and design philosophies (Table 1). In this section we review the full landscape, with particular depth on the models that have been directly applied to or benchmarked on siRNA prediction tasks.

**Table 1.** RNA foundation models surveyed in this review and their current status in siRNA prediction

Model	Year	Parameters	Training data	Arch.	Key innovation	Used in siRNA
RNA-FM	2022	100M	23.7M ncRNA	BERT	Self-supervised MLM	**Yes**
mRNA-FM	2022	100M	45M mRNA CDS	BERT	mRNA-specific	Indirect
UNI-RNA	2023	25M–400M	1B all RNA	BERT	Largest corpus	No
RNA-MSM	2024	*∼* 100M	Rfam MSAs	MSA-Trans.	Co-evolutionary	No
RNAErnie	2024	105M	ncRNA	BERT	Motif masking	No
ERNIE-RNA	2024	86M	ncRNA	BERT	Structure-enhanced	No
UTR-LM	2024	1M	5$^{\prime }$ UTRs	BERT	UTR-specific	No
RNAGenesis	2024	1B	Multimodal	Enc-Dec	RNATx-Bench	Benchmark
AIDO.RNA	2024	1.6B	Diverse RNA	BERT	SOTA on 24/26 tasks	No
Evo 1	2024	7B	Prokaryotic genomes	StripedHyena	DNA (genomic)	No
RiNALMo	2025	650M	36M ncRNA	BERT	RoPE, FlashAttn2	No
HydraRNA	2025	N/R	mRNA + ncRNA	Trans+SSM	Hybrid arch.	No
Orthrus	2026	Small	Mature RNA (isoforms + orthologs)	Mamba/SSM	Contrastive; low-data	No (plausible)
Evo 2	2026	7B/40B	9.3T nt, all domains	StripedHyena 2	DNA/RNA/protein	No

Of the models listed, only RNA-FM has been directly employed in published siRNA predictors, and RNAGenesis contributes a therapeutic-RNA benchmark; the remainder have not, to our knowledge, been applied to siRNA prediction and are included as prospective resources whose relevance is discussed as such in the text. The entry marked ‘plausible’ has not been used for siRNA but is a strong, specifically argued candidate (Orthrus, see text). ‘Used in siRNA’ indicates direct use of a model’s embeddings in a published siRNA predictor. Models are ordered by publication date.

### RNA-FM and mRNA-FM: The backbone of current siRNA prediction

RNA-FM [21], originally described on arXiv and later published alongside the RhoFold+ structure prediction system in Nature Methods [38], introduced a 12-layer BERT-style transformer encoder pretrained on ∼23.7 million noncoding RNA sequences from the RNAcentral database. Using masked language modeling with 15% random token masking, RNA-FM learns 640-dimensional contextual embeddings per nucleotide. These embeddings encode rich information about local sequence motifs, base-pairing propensities, evolutionary conservation patterns, and even three-dimensional proximity signals, all without any experimental labels during pretraining. Among all RNA foundation models, RNA-FM has had the most direct impact on siRNA design. Both OligoFormer [23] and AttSiOff [24] use RNA-FM embeddings as core input representations, and [39] also incorporates them alongside one-hot encoding and thermodynamic features. In each case, ablation studies show that removing RNA-FM embeddings substantially degrades performance, confirming that the pretrained representations carry information that is both relevant to siRNA efficacy and nonredundant with other feature types. The model’s success in this context demonstrates the power of transfer learning: self-supervised pretraining on a large, diverse RNA corpus captures sequence regularities that transfer effectively to the extremely data-scarce siRNA prediction setting.

A direct extension, mRNA-FM, was trained exclusively on 45 million mRNA coding sequences (CDS) and is specifically designed to capture information unique to protein-coding transcripts. Because siRNA targets are typically mRNA molecules, mRNA-FM embeddings may provide complementary information about target-specific context that the noncoding RNA **(ncRNA)**-focused RNA-FM does not capture as strongly, though direct comparisons in siRNA prediction tasks remain limited.

### Scaling RNA language models: RiNALMo and UNI-RNA

Whether scaling RNA language models yields the same systematic improvements observed in protein and natural language models has been addressed by two major efforts.

RiNALMo [22], published in Nature Communications, is the largest dedicated RNA language model available with published weights, at 650 million parameters pretrained on 36 million noncoding RNA sequences. The architecture incorporates several modern techniques: rotary position embeddings (RoPE) enable improved length generalization beyond training sequence lengths, the SwiGLU activation function replaces standard ReLU, and FlashAttention-2 provides the computational efficiency needed to train at this scale. RiNALMo demonstrates state-of-the-art performance across multiple RNA structure and function prediction benchmarks, and critically, it generalizes to RNA families not encountered during training. This generalization capability is directly relevant to siRNA design, where models must perform on novel target sequences. [40] evaluated RNA-FM, RiNALMo, and several other RNA language models on secondary structure prediction, finding that while larger models generally performed better, the advantage depended on the RNA family and evaluation protocol.

UNI-RNA [41] took a different approach to scaling: rather than increasing model parameters, it trained an ensemble of models ranging from 25 million to 400 million parameters on approximately one billion RNA sequences drawn from five sequence databases spanning both coding and noncoding RNAs, with redundancy removed by MMseqs2 clustering. This universal pretraining strategy aims to capture the broadest possible diversity of RNA sequence patterns. While UNI-RNA has not yet been directly applied to siRNA efficacy prediction in a published study, its embeddings represent a potentially valuable resource for future siRNA models given the breadth of sequence diversity captured during pretraining.

### Domain-aware pretraining: RNAErnie and ERNIE-RNA

Two models from distinct research groups introduced the idea of injecting domain-specific biological knowledge into the pretraining process itself, rather than relying solely on the model to discover relevant patterns from raw sequences.

RNAErnie [42], published in Nature Machine Intelligence, introduces motif-level random masking alongside standard base-level and subsequence-level masking during pretraining. By incorporating known RNA biological motifs as structural priors in the masking strategy, RNAErnie encourages the model to learn representations that are aware of biologically meaningful sequence patterns from the start. The model additionally tokenizes RNA types (miRNA, long noncoding RNA (lncRNA), ribosomal RNA, etc.) as special tokens appended to input sequences, enabling a type-guided fine-tuning strategy where the predicted RNA type modulates the embedding during downstream adaptation.

ERNIE-RNA [43] takes a complementary approach by incorporating structure-enhanced representations during pretraining. This model augments the standard masked language modeling objective with auxiliary tasks that encourage the learned representations to encode structural information explicitly, rather than relying on the model to implicitly capture structure from sequence alone. Both models demonstrate that incorporating domain knowledge during pretraining can improve downstream performance, a finding that connects to the broader theme in this review that biology-informed approaches consistently outperform purely data-driven alternatives.

### Therapeutic-focused models: RNAGenesis and AIDO.RNA

The most recent generation of RNA foundation models has been explicitly designed with therapeutic applications in mind, reflecting the growing clinical importance of RNA-based medicines.

RNAGenesis [44] presents a unified architecture at one billion parameters, combining a pretrained encoder with a latent diffusion decoder and multimodal fusion modules. Its most significant contribution for the siRNA community is RNATx-Bench, a dedicated therapeutic RNA benchmark comprising over 100 000 experimentally validated RNAs across six modalities: antisense oligonucleotides (ASOs), siRNAs, short hairpin RNAs, circular RNAs, aptamers, and human UTR variants. On the BEACON benchmark of 13 RNA tasks, RNAGenesis achieves top performance on 11, with siRNA prediction AUROC exceeding 0.8, outperforming RNA-FM and other foundation models on therapeutic prediction tasks. RNATx-Bench addresses a critical gap by providing standardized evaluation protocols that enable fair cross-method comparison specifically for therapeutic RNA applications.

AIDO.RNA [45], presented at the NeurIPS 2024 Workshop on AI for New Drug Modalities, at 1.6 billion parameters is the largest RNA foundation model to date and achieves state-of-the-art results on 24 out of 26 tasks in its RNA sequence understanding benchmark. The benchmark spans nine task categories including structure prediction, function prediction, and mRNA-related tasks relevant to vaccine design. While direct siRNA efficacy prediction results are not the primary focus, AIDO.RNA’s strong performance on diverse RNA understanding tasks and its explicit design for drug modalities make it a potentially powerful backbone for future siRNA prediction systems.

### Specialized and emerging models

Several additional foundation models address specific RNA subtypes or employ novel architectural approaches that may have implications for siRNA design.

UTR-LM [46], published in Nature Machine Intelligence, is a specialized 5$^{\prime }$ UTR language model trained specifically for decoding untranslated regions of mRNA and predicting their functional properties. While not directly applicable to siRNA efficacy prediction, UTR-LM is relevant to understanding the mRNA context surrounding siRNA target sites, as 5$^{\prime }$ UTR structure and regulatory elements can influence mRNA accessibility and translation status.

RNA-MSM [47] takes a fundamentally different input approach by using multiple sequence alignments (MSAs) rather than single sequences. This MSA-based strategy captures co-evolutionary information across homologous RNA families, analogous to how MSA-based protein models capture residue co-variation. The co-evolutionary signal may be relevant for siRNA target site selection, where conserved regions across species or transcript variants may indicate functionally important and structurally constrained sites, though this connection is plausible rather than demonstrated and has not been tested for siRNA.

Evo 1 [48] (Science 2024) and Evo 2 [49]( Nature 2026) are genome-scale DNA foundation models; Evo 1 has 7 billion parameters, and Evo 2 was released in 7- and 40-billion-parameter variants. These models are not RNA-specific and have not been applied to siRNA prediction. Rather than infer broad relevance from genomic context alone, we defer their treatment to the ‘DNA foundation models: relevance and transferable lessons’ section, where their specific transferable lessons, long-context modeling and variant-effect prediction, are developed.

HydraRNA [50], published in Genome Biology, introduces a hybrid architecture combining transformer attention with structured state space models (SSMs) for processing full-length RNA sequences, addressing the length limitations of purely transformer-based approaches. PlantRNA-FM [51], published in Nature Machine Intelligence, provides plant-specific RNA representations relevant to agricultural RNAi applications. Finally, [52] developed an RNA foundation model specifically for therapeutic discovery, demonstrating that pretrained RNA representations can identify disease mechanisms and candidate therapeutics. Orthrus [53] is a notable recent departure in both objective and architecture: a Mamba-based mature-RNA foundation model pretrained not by masked-token prediction but by a self-supervised contrastive objective that maximizes embedding similarity between biologically related transcripts, namely splice isoforms across 10 organisms and orthologous transcripts across >400 mammalian species. Two properties make Orthrus a concrete rather than speculative candidate for siRNA prediction. First, it learns representations of mature mRNAs, precisely the molecules siRNAs target, addressing the same need that motivates mRNA-FM but with an objective tailored to functional similarity. Second, and most relevant to the data-scarcity problem that recurs throughout this review, Orthrus reaches state-of-the-art mRNA property prediction in very low-data regimes, reportedly with as few as a few dozen labeled fine-tuning examples; an embedding that transfers from tens of examples is well matched to siRNA efficacy datasets, and especially to the small chemically modified datasets of the ‘Chemical modification’ section. Benchmarking Orthrus embeddings against RNA-FM in an existing siRNA pipeline is therefore among the most promising of the drop-in-replacement experiments proposed in this review.

### DNA foundation models: Relevance and transferable lessons

The foundation models reviewed above are trained on RNA, but a parallel and faster-scaling line of genome-scale DNA language models warrants explicit comparison, since siRNA targets are ultimately encoded in genomic DNA. DNABERT and its successor DNABERT-2 [54], the Nucleotide Transformer family [55], and the long-context Evo models learn from genomic rather than mature-transcript sequence. The Evo entry deserves precision, as the two releases differ substantially. Evo 1 [48] is a 7-billion-parameter StripedHyena model trained on prokaryotic and bacteriophage genomes with a context of roughly 131 kilobases. Evo 2 [49] was released in 7-billion and 40-billion-parameter variants trained on ∼9.3 trillion nucleotides spanning bacteria, archaea, eukarya, and bacteriophage across >100 000 genomes, with a one-megabase context window at single-nucleotide resolution and generalist prediction and design capabilities across DNA, RNA, and protein.

We did not adopt a genome-scale DNA model as a primary embedding source for siRNA prediction, for three reasons. First, siRNA efficacy is governed by mature-transcript and duplex-level biophysics, namely RISC loading, seed pairing, and target-site accessibility, which mature-RNA and ncRNA models such as RNA-FM and RiNALMo represent more directly than models trained on full genomic context. Second, genomic DNA models encode intronic and regulatory sequence that is absent from the spliced mRNA an siRNA actually engages, so their representations are only partially aligned with the target. Third, no published siRNA predictor has yet used a genome-scale DNA model as its embedding source, so any such use would be exploratory. It is worth noting, however, that this review already contains a worked example of useful DNA-to-siRNA transfer: BERT-siRNA [25] inherits DNA-BERT weights and, after fine-tuning, recovers the 5$^{\prime }$ guide-end strand-selection signal, demonstrating that the shared nucleic-acid alphabet permits productive cross-domain transfer even from a DNA pretraining source.

Three lessons from the DNA foundation-model literature are nonetheless directly relevant to siRNA design. First, long-context genomic architectures, exemplified by Evo 2’s one-megabase window and paralleling the transformer plus state-space hybrid of HydraRNA, point toward models that can reason over full-length transcripts and surrounding regulatory context, which is pertinent to accessibility-aware and isoform-aware target-site selection. Second, Evo 2’s single-nucleotide-resolution variant-effect prediction is the genomic analogue of the in-silico mutagenesis used to generate and validate siRNA saliency maps, and genomic variant scores could be used to prioritize or avoid polymorphic target sites, a capability that connects to the population-aware design direction in the ‘Future directions’ section. Third, the efficiency gains of state-space and convolutional architectures over dense attention at genome scale inform how RNA models themselves might be scaled. Taken together, DNA foundation models are best viewed as complementary providers of regulatory context, variant effects, and long-range structure rather than as replacements for mature-RNA embeddings, and a systematic benchmark of a genomic model such as Evo 2 against RNA-FM and RiNALMo embeddings inside an existing siRNA pipeline is a concrete and high-value open experiment.

### Comparative landscape and impact on siRNA design

Table 1 summarizes the key characteristics of available RNA foundation models. The models differ substantially in scale (from tens of millions to billions of parameters), training data (ncRNA, mRNA, genomic sequences, or combinations), architecture (encoder-only BERT-style, encoder–decoder, or hybrid), and the degree to which domain knowledge is incorporated during pretraining versus fine-tuning.

A candid caveat about scope is warranted. Of the models in Table 1, only RNA-FM has been directly applied in published siRNA predictors, and RNAGenesis contributes a benchmark rather than an embedding used inside an siRNA model; the remaining models have not, to our knowledge, been used for siRNA prediction. We survey them because a map of the representational landscape is useful for readers planning future work, but their relevance to siRNA is prospective rather than demonstrated, and the strength of that prospect varies. The strongest cases are models whose training data or design align with siRNA targets: mRNA-FM, trained on the coding transcripts that siRNAs engage; RiNALMo and AIDO.RNA, whose scale and benchmarked generalization make them plausible drop-in replacements for RNA-FM embeddings; Orthrus, whose mature-RNA representations and low-data fine-tuning efficiency suit the small siRNA datasets (see the ‘Chemical modification’ section); and RNAGenesis, whose RNATx-Bench includes siRNA tasks. For others, including RNA-MSM, UTR-LM, and the genome-scale DNA models Evo 1 and Evo 2, the connection is more indirect, and we flag these as speculative. The specific rather than generic lessons that genome-scale DNA models offer siRNA design are developed in the ‘DNA foundation models: Relevance and transferable lessons’ section, not asserted as broad relevance here.

The foundation-model landscape is moving quickly, and the two most recent additions here are illustrative: ESM-3 for proteins and Orthrus for RNA show that the current frontier emphasizes multimodal generation and biologically informed pretraining objectives over scale alone.

### Lessons from protein language models: the generative paradigm

RNA foundation models for siRNA prediction build on the more mature protein language modeling trajectory, where autoregressive and masked models have achieved not just prediction but *de novo* sequence generation. This protein LLM paradigm provides essential context for evaluating RNA foundation models.

ProGen [56], a 1.2-billion-parameter autoregressive transformer trained on 280 million protein sequences from over 19 000 families, showed that language models can generate protein sequences with predictable functions. Using control tags specifying protein family, biological process, and molecular function, ProGen generates sequences that, when fine-tuned on specific enzyme families, produce artificial lysozymes with catalytic efficiencies comparable to natural enzymes despite sequence identity as low as 31%. Strikingly, this shows that generative protein language models have learned sufficient biochemical knowledge from sequence data alone to produce functional molecules, not merely plausible-looking sequences.

ProtGPT2 [57], a 738-million-parameter GPT-2-style decoder trained on 45 million UniRef50 sequences, showed that unsupervised autoregressive models generate sequences with natural amino acid propensities, with 88% of generated sequences predicted to be globular (consistent with natural proteins). Crucially, ProtGPT2 sequences explore regions of protein space not represented in existing databases while maintaining structural plausibility as confirmed by AlphaFold2 structure prediction. ProGen2 [58] scaled this approach to 6.4 billion parameters and conducted systematic scaling analysis, finding that model perplexity improves consistently with scale but that zero-shot fitness prediction peaks at 764 million parameters before declining, suggesting that larger models may overfit to phylogenetic biases in training data. This nonmonotonic scaling behavior is an important cautionary finding for the RNA foundation model community.

The most recent advance in this line, ESM-3 [59], reasons jointly over protein sequence, structure, and function as discrete token tracks within a single multimodal masked generative transformer scaled to 98 billion parameters, and demonstrates conditional generation of functional proteins far from any natural sequence, including a novel fluorescent protein roughly 58% identical to its closest natural relative. ESM-3 sharpens rather than merely updates the lesson drawn from ProGen and ProtGPT2: the frontier of biomolecular modeling is now multimodal and generative. The analogous capability for siRNA, conditional generation of a duplex jointly specified by target sequence, structural context, and desired functional and modification properties, remains entirely unrealized, as discussed in the ‘From ranking to generation: Limitations of the prediction paradigm and the case for generative siRNA design’ section.

These protein LLM successes establish several principles directly relevant to siRNA prediction. First, self-supervised pretraining on large sequence corpora captures biochemically meaningful patterns that transfer to downstream tasks, even when the pretraining objective (next-token or masked-token prediction) bears no explicit relationship to the downstream task. This underlies the use of RNA-FM embeddings in OligoFormer and AttSiOff. Second, the generative capability of protein LLMs raises whether similar generative approaches could be applied to siRNA design: rather than enumerating all possible candidates by sliding window and ranking them by predicted efficacy, a generative RNA model could directly propose high-efficacy sequences conditioned on a target mRNA. No published work has yet demonstrated this generative approach for siRNA, but the protein LLM precedent suggests it is feasible, representing a paradigm shift from prediction to generation.

Table 2 summarizes the protein language models whose architectural innovations and generative capabilities provide direct precedents for RNA foundation model development.

**Table 2.** Protein language models providing architectural precedents for RNA foundation models

Model	Year	Params	Training data	Architecture	Key capability	Therapeutic relevance
*Protein language models*						
ProGen	2023	1.2B	280M proteins	Autoregressive	Functional enzyme generation	Direct: generates therapeutics
ProtGPT2	2022	738M	45M proteins	Autoregressive	Novel fold exploration	Explores unseen protein space
ProGen2	2023	6.4B	49M+ proteins	Autoregressive	Fitness prediction, scaling	Scaling laws for bio-LLMs
ESM-2 [60]	2023	15B	250M proteins	Masked LM	Structure prediction	Embeddings for downstream
ESM-3 [59]	2025	98B	Seq/struct/func tokens	Multimodal masked generative	Multimodal conditional generation	Programmable design of novel functional proteins (esmGFP)

The generative capabilities demonstrated by ProGen, ProtGPT2, and ProGen2 motivate the unexplored direction of generative siRNA design.

### Implications for siRNA Prediction

For siRNA design specifically, the most impactful observation from this landscape is that RNA-FM, despite being neither the largest nor the most recent model, remains the most widely adopted in published siRNA prediction systems. This likely reflects both its early availability and the fact that its 640-dimensional embeddings have been empirically validated in the siRNA context by OligoFormer, AttSiOff, and DeepSipred. However, this also is an opportunity: none of the larger or more recent models (RiNALMo, UNI-RNA, RNAGenesis, AIDO.RNA) have been systematically evaluated as drop-in replacements for RNA-FM in siRNA prediction pipelines. Given the consistent scaling improvements observed across these models on other RNA tasks, it is plausible that substituting RNA-FM embeddings with those from RiNALMo or RNAGenesis could yield further improvements in siRNA efficacy prediction. This systematic comparison represents a concrete, high-value near-term experiment that the community should prioritize.

The protein LLM trajectory also suggests a more ambitious future direction: moving from siRNA efficacy *prediction* (ranking pre-enumerated candidates) to siRNA sequence *generation* (directly proposing optimal sequences conditioned on target context). While the standard sliding-window-and-rank workflow has been effective, it is fundamentally constrained to the space of contiguous subsequences within a target mRNA. A generative approach, inspired by ProGen’s conditional generation with control tags, could potentially explore chemical modification patterns, noncanonical base compositions, or even chimeric designs that the enumerate-and-rank paradigm cannot access. The prerequisites for this shift, large-scale pretrained RNA models, established evaluation benchmarks, and thermodynamic validation tools, are all now available; the missing component is a generative siRNA model itself.

More broadly, foundation models address the fundamental data-scarcity problem at the heart of siRNA prediction. Even the largest curated efficacy datasets contain only thousands of validated siRNAs, while foundation models learn from tens of millions to billions of RNA sequences. The pretrained embeddings capture structural, evolutionary, and motif information that is mechanistically relevant to siRNA function but prohibitively expensive to learn from siRNA data alone. Ablation studies across OligoFormer, AttSiOff, and DeepSipred consistently confirm that foundation model embeddings carry information that is nonredundant with both hand-crafted thermodynamic features and sequence-level encodings, validating the hybrid approach as the dominant design pattern for state-of-the-art siRNA prediction.

### Limitations and capability boundaries of RNA foundation models

The preceding sections establish the value of RNA foundation models, but a critical review must also delimit what they cannot do, and the most consequential boundary is a representational one. RNA-FM, RiNALMo, ERNIE-RNA, and the other models in Table 1 are pretrained on natural RNA over the canonical four-letter alphabet, and their tokenizers contain no symbols for 2$^{\prime }$-O-methyl, 2$^{\prime }$-fluoro, phosphorothioate, locked nucleic acid, glycol nucleic acid (GNA), or 5$^{\prime }$-(E)-vinylphosphonate. A chemically modified nucleotide is therefore either collapsed to its canonical parent, which discards the modification entirely, or treated as out of vocabulary. Because every FDA-approved therapeutic siRNA is heavily modified, the embeddings these models produce describe only the unmodified sequence skeleton and are blind to the chemistry that governs nuclease resistance, immunostimulation, and modified-duplex thermodynamics. This is precisely why ENsiRNA and Cm-siRPred attach explicit modification encodings rather than relying on foundation-model embeddings: the foundation models cannot represent the inputs that matter most in a therapeutic context.

Beyond the alphabet lies a deeper distributional mismatch. Pretraining corpora are dominated by natural RNA families such as ribosomal, transfer, and long noncoding RNAs that carry strong evolutionary conservation signal, whereas a therapeutic siRNA is a short synthetic 21-mer with no evolutionary history and chemistries absent from nature. Much of what foundation models exploit, namely cross-family conservation and co-evolutionary covariation, is largely irrelevant to a designed duplex, so the transfer benefit is realized mainly through generic local-sequence and secondary-structure regularities rather than therapeutic-specific signal. Reported area under the receiver operating characteristic curve (AUC) gains can therefore overstate the models’ grasp of therapeutic siRNA behavior, and they degrade precisely where modification chemistry and assay context dominate, consistent with the leakage effects identified by [26]. This therapeutic RNA distribution shift, between the natural ncRNA on which models are trained and the modified synthetic oligonucleotides on which they are deployed, has not been systematically quantified and is an important gap.

A modification-aware RNA foundation model would require three things the field largely lacks: an expanded chemical alphabet or, more fundamentally, a move from sequence tokens to atom-level or graph-level molecular representations capable of encoding arbitrary modifications; large pretraining corpora of modified oligonucleotides at a scale comparable to natural-RNA databases, which do not exist and which the data-scarcity argument of the ‘Future directions’ section addresses directly; and pretraining objectives that capture modified-duplex thermodynamics and protein-interaction geometry rather than masked prediction over canonical bases. The most promising directions sidestep the alphabet limitation through molecular representations: the E(3)-equivariant three-dimensional encoding of ENsiRNA [27], the extended connectivity fingerprints (ECFP) and energy-minimized siRNA/AGO2 structural alignments of [61], and the SMILES-level annotations stored in the siRNAmod database [62] all represent modifications at the chemical rather than the symbolic level. Notably, even frontier genomic models such as Evo 2 [49] operate on canonical nucleotides and do not solve this problem. The bottom line is a genuine capability boundary rather than a minor gap: no modification-aware RNA foundation model currently exists, and building one is among the most consequential open problems for therapeutic siRNA design.

### Quantifying the contribution: classical features versus foundation-model embeddings

A natural question spanning the ‘Classical rules and early machine learning’ and ‘RNA foundation models as pretrained representations’ sections is whether classical thermodynamic and sequence features retain predictive value once foundation-model embeddings are available, or whether the embeddings subsume them. The ablation evidence is unambiguous: classical biophysical features remain nonredundant. The clearest decomposition comes from OligoFormer, whose per-module ablation isolates each contribution on the same data. OligoFormer’s per-module ablation shows that each information source is nonredundant: removing the RNA-FM embeddings or the thermodynamic features each degrades accuracy, and removing the Oligo encoder (the convolution–BiLSTM–transformer stack) degrades it the most, which the OligoFormer authors highlight as evidence of the transformer’s indispensability. (On its headline intra-dataset benchmark the full model reaches an AUC of 0.862 on Huesken, Table 10.) Two points follow. First, both the learned embeddings and the hand-computed thermodynamic features contribute beyond what the other provides, even though the embeddings are learned from tens of millions of sequences and the thermodynamic features encode a handful of nearest-neighbour energies; end asymmetry, in particular, behaves as the single most durable signal in the field, exactly as it did in the Reynolds and Shabalina era. Second, the architecture itself contributes the largest single effect, indicating that the value of the embeddings is realized through, not independently of, the attention mechanism that integrates them with physical priors.

This pattern is not specific to OligoFormer. AttSiOff and DeepSipred both report that ablating either the foundation-model embeddings or the thermodynamic and prior-knowledge features degrades performance, confirming that the two information sources are complementary rather than substitutable. siRNADiscovery, which forgoes foundation-model embeddings entirely in favor of one-hot encodings with positional context, instead finds that its thermodynamic co-fold interaction nodes and AGO2-interaction features are the critical components, again placing biophysical information at the centre of the prediction. ENsiRNA fuses three-dimensional geometry, language-model embeddings, thermodynamics, and modification tags, improving Pearson correlation by roughly 13% over prior methods, with no single modality dominating. Table 3 summarizes this evidence. The consistent message is that the modern hybrid models did not replace the classical features distilled in the ‘Classical rules and early machine learning’ section; they re-weighted and contextualized them with learned representations, which is the continuity that links the two method eras.

**Table 3.** Ablation-based contribution of classical thermodynamic and sequence features versus RNA foundation-model (FM) embeddings in representative hybrid siRNA efficacy predictors

Hybrid model	Reported result	*Δ* w/o thermo. features	*Δ* w/o FM embeddings	Contribution pattern
OligoFormer [23]	AUC 0.862 (Huesken)	degrades	degrades	Both nonredundant; Oligo-encoder (transformer) removal is the largest single effect
DeepSipred [39]	10-fold CV	degrades	degrades	One-hot, RNA-FM, and Gibbs free energy each nonredundant
AttSiOff [24]	—	degrades	degrades	RNA-FM embeddings and thermodynamic priors jointly required
ENsiRNA [27]	*+∼* 13% PCC	—	—	3D geometry, FM embeddings, thermodynamics, and modification tags fused; no single modality dominant
siRNADiscovery [26]	AUC 0.874 (siRNA-split)	co-fold/AGO2 nodes critical	FM not used (one-hot + positional)	Thermodynamic interaction features dominate without FM embeddings

Quantitative per-module deltas are available only for OligoFormer; for the other models the reported direction of the ablation effect is summarized. Across all models, removing thermodynamic features degrades performance even when FM embeddings are retained, confirming that classical biophysical features remain nonredundant in the foundation-model era.

## Transformer-based architectures for knockdown efficacy prediction

A key distinction frames this section: the methods reviewed here address siRNA *knockdown efficacy prediction*, not *de novo* sequence design. Given a candidate siRNA and its mRNA target context, these models predict silencing effectiveness as a percentage inhibition or continuous score. The practical workflow enumerates all 21-nucleotide candidates along a target mRNA by sliding window, ranks them by predicted knockdown, and filters for off-target safety. This prediction framing shapes evaluation metrics (Pearson correlation, AUC), training data (measured knockdown values), and architecture (fixed-length input to scalar output, no generative component needed). Transformer architectures now dominate this task, using self-attention to model dependencies across guide and target while integrating diverse feature sources through learned weights.

### OligoFormer: Integrating thermodynamics, foundation models, and attention

OligoFormer [23] is the current state of the art in siRNA knockdown efficacy prediction and exemplifies the hybrid approach that has proven most effective across the field. Rather than relying on any single information source, OligoFormer integrates three synergistic modules into a unified prediction framework, each addressing a different aspect of siRNA biology.

The thermodynamic feature module computes detailed free-energy profiles from the siRNA duplex and its target context: overall duplex stability (*Δ G*), position-specific stabilities across 2-nucleotide sliding windows that capture local stability variation along the duplex, and the critical end asymmetry ($\Delta \Delta G$) that governs strand selection during RISC loading. These features directly encode two decades of biophysical understanding about the RNAi mechanism, from the Reynolds rules through the Shabalina thermodynamic models. The RNA-FM embedding module extracts 640-dimensional contextual representations from the pretrained RNA foundation model, capturing evolutionary and structural patterns that extend far beyond what siRNA-specific training data could teach. Finally, the core architectural component is a transformer encoder that processes concatenated guide and target sequence representations through a pipeline of 2D convolution (for local pattern extraction), bidirectional LSTM (for sequential context), and a two-layer multihead transformer encoder that learns to weight and integrate all feature sources through attention.

The training strategy is notable for its careful handling of dataset heterogeneity. OligoFormer trains on 3,714 siRNAs drawn from nine experimental datasets, each collected under different cell lines, transfection conditions, readout methods, and measurement timescales. Dataset-specific normalization prevents any single experimental protocol from dominating the learned representations, and cross-dataset evaluation confirms robust generalization rather than overfitting to any single experimental context. In OligoFormer’s inter-dataset evaluation, where the model is trained on one dataset and tested on another, this yields an average AUC improvement of ∼9% and an F1 improvement of 10.7% over previous methods; intra-dataset margins on any single benchmark are correspondingly smaller (Table 10).

The interpretability analyses accompanying OligoFormer are among the most detailed in the siRNA prediction literature and provide critical evidence that the model learns biologically meaningful representations rather than exploiting dataset artifacts. Attention maps demonstrate that the transformer learns to focus on the seed region (positions 2–8 of the guide strand) and cleavage-proximal positions (around positions 10 and 11), both of which are mechanistically critical for siRNA function. Saliency maps generated via gradient backpropagation through the full network reveal that A and G nucleotides at positions 1 and 2 and U at positions 18 and 19 exhibit the highest importance scores for efficacy prediction, patterns that are consistent with the empirically established design rules of [8] and the asymmetry principles of [9]. Ablation studies provide quantitative evidence for the nonredundancy of each module: removing the thermodynamic features or the RNA-FM embeddings each degrades performance, and removing the Oligo encoder (the transformer stack) produces the largest drop, from a full-model AUC of 0.862 on Huesken (Table 10). These ablation results are perhaps the clearest evidence in siRNA prediction that physics-based features, pretrained representations, and learned attention provide genuinely complementary information.

### AttSiOff: Joint efficacy and off-target prediction

While most models treat knockdown efficacy and off-target risk as separate prediction tasks, AttSiOff [24] uniquely couples both objectives within a single architecture. The system combines multihead self-attention over RNA-FM embeddings with a suite of prior-knowledge features including thermodynamic descriptors and structural properties. The attention mechanism processes the concatenated guide and local mRNA context, learning to weight positions according to their contribution to both efficacy and specificity.

What distinguishes AttSiOff from other efficacy predictors is its integrated pipeline approach. Beyond the core prediction module, the system includes an mRNA searching package that automatically retrieves the latest mature mRNA sequences from genome databases for a given gene name and enumerates all candidate siRNAs by sliding window. An off-target filter then computes the number of potential seed-matched binding sites across the transcriptome for each candidate, providing a specificity score alongside the efficacy prediction. This end-to-end pipeline, from gene name to ranked candidates with both efficacy and off-target scores, directly addresses the practical workflow that experimental researchers follow. The clinical relevance of AttSiOff’s predictions has been validated against five FDA-approved siRNA drugs (targeting TTR, ALAS1, PCSK9, and HAO1), where the predicted inhibitions rank near the top among all possible candidates for their respective gene targets (within the top 1%–12% of candidates), suggesting that the model captures features relevant to therapeutic-grade knockdown.

### BERT-siRNA: transfer learning across nucleic acid domains

BERT-siRNA [25] explores an alternative strategy for addressing the limited training data problem: rather than using RNA-specific foundation model embeddings as input features, it adapts the BERT architecture itself through a two-stage transfer learning process. The model first inherits pretrained weights from DNA-BERT, a bidirectional encoder trained on large DNA sequence corpora, then fine-tunes on siRNA efficacy data. This cross-domain transfer leverages the structural similarities between DNA and RNA sequences (both are nucleic acids with overlapping alphabets and shared biochemical properties) to bootstrap learning from a much richer pretraining source.

The attention visualizations produced by BERT-siRNA provide independent evidence that transformer models learn biologically meaningful features for siRNA prediction. Even though the model was pretrained on DNA rather than RNA, the fine-tuned attention patterns show biologically meaningful focus on the $5^{\prime }$ end of the guide strand, consistent with the known importance of this region for strand-loading asymmetry during RISC assembly. This cross-domain interpretability finding suggests that the sequence patterns governing strand selection are robust enough to be partially captured even from a related but distinct pretraining domain.

### DeepSilencer, deepsipred, and data-efficient approaches

The chronic scarcity of experimentally validated siRNA knockdown data has motivated several architectures that focus specifically on maximizing prediction performance under data-limited conditions. DeepSilencer [63] introduces Selective Pair Sampling, a dynamic random pairing mechanism that creates informative training pairs from existing data points, effectively augmenting the training signal without requiring additional experimental measurements. Combined with a Tri-Task Learning Framework that enables multidataset alignment (learning shared representations across heterogeneous experimental sources), DeepSilencer achieves state-of-the-art performance on the challenging Mixset benchmark using only sequence and basic physicochemical properties, without requiring any pretrained foundation model embeddings. This result is significant because it shows that careful training-strategy innovation can partially compensate for the absence of large-scale pretraining, potentially making effective siRNA prediction accessible to research groups without the computational resources to deploy large foundation models. It also makes DeepSilencer an important counterexample to the common claim that foundation-model embeddings are necessary for state-of-the-art performance: on Mixset it matches or exceeds OligoFormer (AUC 0.858 versus 0.845) using only sequence and physicochemical features. The robust generalization is therefore the integration of physical priors with learned representations, which DeepSilencer also embodies, rather than the use of pretrained embeddings specifically (see the ‘Conclusions’ section).

39] takes a complementary approach by combining multiple feature modalities through multikernel convolutions: one-hot sequence encoding and RNA-FM embeddings provide learned representations, Gibbs free energy calculations provide thermodynamic grounding, and established design criteria from the literature provide expert knowledge. The multikernel architecture uses convolutions of different sizes to detect sequence motifs at various scales, from dinucleotide preferences to longer-range compositional patterns. Benchmarked via 10-fold cross-validation on large public datasets, DeepSipred confirms that convolutional architectures remain competitive when the feature engineering is thoughtful and the integration of pretrained representations is done carefully.

Recent work by [64] provides a valuable reality check for the field by demonstrating that classical machine learning with careful feature engineering can achieve surprisingly strong knockdown efficacy prediction. Using intrinsic antisense sequence features (sequence composition, regulatory motifs, thermodynamic parameters, and structural complexity descriptors) with support vector regression, they achieve *R = 0.719* and $R^2 = 0.516$ on the standard Huesken benchmark. This performance, while below the best deep learning methods, suggests that the field may sometimes overvalue architectural complexity relative to the contribution of thoughtful feature design, and that the marginal gains from increasingly sophisticated architectures should be evaluated against the simpler baselines that careful feature engineering can achieve.

### Training objectives: from pointwise prediction to pairwise ranking

The standard training objective for knockdown efficacy prediction treats each siRNA independently, minimizing the mean squared error (or cross-entropy for classification) between predicted and measured knockdown values. However, this pointwise formulation does not match how practitioners actually use prediction models: in practice, the goal is to rank many candidate siRNAs for a single target gene and select the top few for synthesis, so relative ordering matters more than absolute prediction accuracy.

[65] address this mismatch by formulating siRNA prediction as a preference-learning problem using ranking-based transformer objectives. Their approach constructs preference pairs from within-gene candidate sets and applies debiased training to reduce the influence of dataset-specific biases that can distort rankings. While still an emerging direction, this training paradigm is better aligned with the practical screening workflow and may produce models that are more useful for experimental decision-making even if their pointwise correlation metrics are comparable to standard approaches.

### From ranking to generation: limitations of the prediction paradigm and the case for generative siRNA design

The methods in this section and the next share a single paradigm: enumerate every contiguous candidate along the target mRNA by sliding window, score each with a trained predictor, and rank. This workflow is effective, but a critical review should confront four limitations it rarely acknowledges. First, it is structurally confined to contiguous subsequences of the target, and so cannot reach chimeric designs, noncanonical base compositions, or, most importantly, chemical-modification patterns, all of which lie outside the enumerable space entirely. Second, it optimizes a proxy, predicted efficacy, and therefore inherits every weakness of the underlying predictor: the leakage that has inflated benchmarks [26], the assay-specific confounds that can invalidate cross-dataset transfer, and the absence of calibrated uncertainty discussed in the ‘Future directions’ section. Third, the standard pointwise training objective is misaligned with the decision that actually matters, which is a within-gene ranking; relative ordering, not absolute accuracy, determines which candidates are synthesized, a mismatch that the preference-learning formulation of [65] begins to address. Fourth, efficacy, off-target risk, and modifiability are handled as sequential filters rather than joint objectives, so the paradigm cannot natively trade them off against one another.

A generative approach would invert this logic: rather than scoring a fixed candidate set, a model would propose high-efficacy sequences directly. The protein-language-model precedent is concrete, as discussed in the ‘RNA foundation models as pretrained representations’ section for ProGen and ProtGPT2, and the closest RNA analogue is RNAGenesis [44], whose latent-diffusion decoder can in principle generate RNA conditioned on context. Yet no published work demonstrates *de novo* generative siRNA design, which is a real and somewhat surprising gap given that the prerequisites, pretrained RNA models, therapeutic benchmarks such as RNATx-Bench, and thermodynamic validators, are all now available. The missing component is a generative siRNA model itself, together with a principled way to score its proposals.

A practical generative system would be conditional, generating duplexes conditioned on the target transcript and on desired properties such as knockdown level, off-target safety, and modification compatibility, either through control tags in the manner of ProGen or through property-guided diffusion, with the existing predictors repurposed as guidance or reward and thereby closing a loop with the active-learning direction of the ‘Future directions’ section. A critical caveat, however, distinguishes the siRNA case from protein design and tempers the enthusiasm. For a 21-mer drawn from a known target, the contiguous-candidate space is small enough that enumerate-and-rank is nearly exhaustive over natural sequences, so the value of generation is not in covering an astronomically large space, as it is for proteins, but specifically in escaping the contiguous-subsequence constraint, namely accessing modification patterns, chemically expanded alphabets, and chimeric or multitarget designs that the ranking paradigm cannot represent. Framed this way, generative siRNA design is less a replacement for prediction than the natural route to modification-aware, multi-objective design, which is exactly where the ranking paradigm reaches its limit.

## Graph neural networks for knockdown efficacy prediction

Unlike transformers, which process siRNA and mRNA as concatenated linear sequences, graph neural networks explicitly encode their interaction topology as structured graphs. Nodes represent nucleotides or functional units, and edges encode spatial, structural, and base-pairing relationships. Message passing propagates information along biologically meaningful pathways rather than relying on positional proximity alone. This inductive bias naturally suits siRNA prediction, where the core event (guide-target base pairing triggering AGO2 cleavage) is inherently graph-structured.

As with the transformer methods, these GNN approaches address knockdown efficacy prediction within the enumerate-and-rank paradigm whose limitations are examined in the ‘From ranking to generation: limitations of the prediction paradigm and the case for generative siRNA design’ section: the models take a candidate siRNA and its mRNA context, build a graph representation of their interaction, and output a predicted knockdown score.

### siRNADiscovery: heterogeneous graphs and rigorous evaluation

siRNADiscovery [26], published in Briefings in Bioinformatics, is the most advanced GNN framework for siRNA knockdown efficacy prediction to date. The model constructs heterogeneous graphs with three distinct node types, each carrying specialized feature sets that capture different aspects of the siRNA/mRNA interaction. siRNA nodes encode the guide strand sequence through one-hot encoding augmented with high-dimensional positional embeddings. mRNA nodes similarly encode the target region with positional context extending beyond the immediate binding site. Interaction nodes, which sit at the interface between siRNA and mRNA nodes in the graph, carry base-pairing probabilities computed from thermodynamic co-fold analysis and RNA/AGO2 interaction probabilities that encode the protein context of the silencing reaction. Message passing across this heterogeneous graph allows the model to learn how features from siRNA, mRNA, and their interaction jointly determine knockdown efficiency, capturing the multi-entity nature of the biological system in a way that concatenated sequence representations cannot.

The choice of one-hot encoding over k-mer representations for node features, while seemingly simple, was validated through careful comparison: the authors demonstrate that one-hot encoding consistently outperforms k-mers (which were used in the earlier GNN4siRNA model) across all performance metrics, because one-hot preserves precise positional information for individual nucleotides while k-mers emphasize local patterns at the expense of position-specific resolution.

However, the paper’s most field-changing contribution is methodological rather than architectural. The authors provide a rigorous analysis demonstrating that previous cross-validation protocols for siRNA prediction suffered from pervasive data leakage. In standard random splitting, siRNAs targeting the same mRNA transcript, or even identical siRNA sequences measured under different conditions, could appear in both training and test partitions. Such leakage means test-set performance partially reflects the model’s ability to memorize training examples rather than to generalize to truly novel siRNAs or targets. The proposed remediation consists of two new splitting protocols: siRNA-split, which ensures that no siRNA sequence in the test set appears in the training set, and mRNA-split, which ensures that no target mRNA in the test set was targeted by any siRNA in the training set. Both protocols use 70:15:15 train/validation/test divisions and are validated across 10 random seeds producing 20 distinct splits, ensuring that reported performance reflects genuine generalization rather than a lucky partition. Under these leakage-aware conditions, the performance gap between different methods narrows substantially, and some approaches that previously appeared superior lose their advantage. This finding has profound implications for the entire field and establishes an evaluation standard that all future work should adopt as a minimum requirement.

### ENsiRNA: three-dimensional geometry and chemical modification

ENsiRNA [27] pushes graph-based knockdown prediction in a new direction by incorporating three-dimensional structural information through E(3)-equivariant geometric graph neural networks. Previous siRNA prediction methods, whether transformer or GNN-based, operated on sequence-level or two-dimensional structural features; ENsiRNA is the first to encode the actual 3D coordinates of nucleotides in the siRNA molecule. The E(3)-equivariant architecture (based on the EGNN framework that has proven successful for molecular property prediction and protein design) ensures that predictions are invariant to arbitrary rotations and translations of the input coordinates, a physically necessary property for any model operating on molecular geometry.

The multimodal integration in ENsiRNA is notably sophisticated. For each siRNA/mRNA pair, the model combines four distinct information sources: sequence features extracted from pretrained RNA language model embeddings, three-dimensional nucleotide coordinate geometry, calculated thermodynamic parameters, and chemical modification encodings that specify which positions carry which types of modifications. This last component is critically important because it makes ENsiRNA the first and currently only method that supports both standard (unmodified) siRNA knockdown prediction and chemically modified siRNA efficacy prediction within a single unified framework. For standard siRNA prediction, ENsiRNA achieves ∼13% improvement in Pearson correlation over previous methods. For modified siRNA prediction, the explicit encoding of modification type and position as node attributes in the geometric graph enables the model to learn how specific modifications at specific positions modulate knockdown efficiency, addressing the practical reality that all therapeutic siRNAs carry extensive chemical modifications.

### GNN4siRNA and GraphSiRNA: foundational contributions

[28] established the conceptual foundation for graph-based siRNA prediction by introducing GNN4siRNA (also referred to as GraphSiRNA), the first model to represent siRNA/mRNA systems as heterogeneous graphs. Using the HinSAGE (Heterogeneous GraphSAGE) architecture, the model creates nodes for siRNAs, target mRNAs, and their interactions, then applies message passing to predict knockdown efficacy. While subsequent architectures (siRNADiscovery with its richer feature engineering and leakage-aware evaluation, ENsiRNA with its 3D geometric encoding) have substantially improved upon GNN4siRNA’s predictive accuracy, the conceptual contribution of this work remains influential. It showed that the multi-entity, graph-structured nature of the siRNA/mRNA/RISC interaction is naturally captured by graph neural networks, opening a new architectural direction for the field that has proven complementary to transformer approaches.

### Comparing graph and transformer architectures for knockdown prediction

The coexistence of GNN-based and transformer-based approaches raises a natural question: which architectural family is better suited for siRNA knockdown efficacy prediction? The evidence suggests that the question is not well-posed, because the two families offer complementary inductive biases that capture different aspects of the underlying biology.

GNNs excel when the prediction task involves structured relationships between entities. The base-pairing topology between guide and target, the secondary structure of the mRNA around the binding site, and the geometric context of the AGO2/siRNA/mRNA ternary complex all have natural graph representations. By encoding these relationships as edges in a heterogeneous graph, GNNs can propagate information along biologically meaningful interaction pathways. The siRNADiscovery results, on mRNA-split evaluation where the model must generalize to entirely new target transcripts, suggest that the graph inductive bias helps the model learn transferable interaction patterns rather than memorizing sequence-specific features.

Transformers, by contrast, excel at modeling long-range dependencies in sequential data and benefit enormously from the rich pretraining ecosystems provided by RNA foundation models. The ability to initialize with pretrained weights from RNA-FM or RiNALMo gives transformer-based approaches a substantial head start on understanding general RNA sequence patterns, while the self-attention mechanism can learn to focus on mechanistically relevant positions (seed region, cleavage site) without explicit guidance. OligoFormer’s attention maps provide direct evidence that transformers can discover biologically meaningful positional patterns from knockdown efficacy data.

The complementarity of these approaches motivates hybrid graph/transformer architectures that remain largely unexplored for siRNA prediction. A natural design would combine a graph tower operating over local secondary-structure windows and base-pairing interaction topology with a transformer tower processing concatenated guide/target sequences enriched by foundation model embeddings, fusing the outputs through cross-attention or late concatenation. Such hybrid architectures have shown promise in related molecular prediction tasks but have not yet been systematically evaluated for siRNA knockdown prediction, representing a concrete opportunity for architectural innovation.

## Biology-informed learning and interpretability

Purely data-driven siRNA prediction models often learn correlations that do not correspond to known biology, especially on small, heterogeneous datasets. Biology-informed approaches address this by encoding mechanistic knowledge as constraints, regularizers, or architectural inductive biases, while interpretability methods evaluate whether learned representations align with established understanding. Together, these threads produce models that are both more accurate and more trustworthy.

### Physics-informed and biology-informed regularization

Physics-informed machine learning [66, 67] has shown that encoding governing equations as differentiable training constraints improves generalization and data efficiency. The central idea transfers to biological applications, though biological priors are softer, more context-dependent, and less precisely quantified than physical equations.

For siRNA specifically, several well-established principles lend themselves to differentiable regularization. Thermodynamic asymmetry can be encoded as a penalty encouraging predictions to correlate with $\Delta \Delta G$ asymmetry. Seed-region composition constraints can penalize high GC content in positions 2–8 (which produces strong off-target effects). GC-content heuristics, immune-motif avoidance, and duplex stability bounds can all be expressed as differentiable terms added to the training objective.

[30] argues that biological systems present unique challenges for informed ML: uncertain and context-dependent priors, heterogeneous data, partial observability, and complex high-dimensional networks. Nevertheless, a biology-informed hybrid model incorporating design principles as soft regularizers demonstrates improved prediction accuracy and better mechanistic alignment of learned attention patterns. This connects siRNA prediction to growing work on biologically interpretable neural networks in cancer genomics [68] and gene regulation [69], where incorporating biological knowledge consistently improves both performance and interpretability.

Even before explicit regularization, integrating calculated thermodynamic features into neural architectures constitutes implicit physics-informed learning. OligoFormer’s thermodynamic module injects domain knowledge through input representation, and the consistent finding that removing these features degrades performance, even with powerful foundation model embeddings, shows that current architectures have not fully learned thermodynamic principles from data alone.

### Interpretability through attention and saliency analysis

Attention-based architectures offer mechanistic interpretability through attention weight matrices and gradient-based saliency maps that quantify each nucleotide position’s contribution to predictions. For siRNA, these analyses extend beyond debugging: saliency maps highlighting the seed region (positions 2–8) can guide experimental mutation priorities and build confidence that models capture known biology.

OligoFormer provides the most detailed saliency analysis. Gradient backpropagation through the full network produces position-by-nucleotide saliency maps across the Huesken, Mixset, and Takayuki datasets, revealing that A and G at positions 1 and 2 and U at positions 18 and 19 receive the highest importance scores, consistent with the nucleotide preferences reported by [70] and the design rules of [8] and [9]. Attention maps show learned focus on the seed region (positions 2–8) and the cleavage-proximal zone (positions 10 and 11). BERT-siRNA provides complementary evidence with attention on the $5^{\prime }$ guide-strand end consistent with strand-loading asymmetry. [71] supply mechanistic ground truth: through thermodynamic analysis with machine learning, they show the seed contains two functionally distinct domains (positions 2–5 essential for off-target, positions 6–8 for both RNAi and off-target), with positions 8–14 showing negative correlation. A persistent concern is whether such explanations faithfully reflect model decisions, since saliency maps can mislead: gradient-based methods can produce similar maps even under randomized weights [72], and attention weights often fail to track feature importance measured by other means [73]. Addressing this question for siRNA specifically, recent work from the present authors’ group [29] proposes a perturbation-based protocol that tests whether mutating high-saliency positions changes model output more than mutating composition-matched control positions, and reports that salient positions tend to cluster in canonical functional regions and that explanation quality can degrade when the measurement assay changes. These results are consistent with the broader and well-established finding that saliency requires explicit faithfulness checks rather than visual inspection alone.

### Interpretability in broader biological context and next steps

These interpretability challenges mirror a broader demand for explainable AI across drug discovery and computational biology. [74] surveys XAI techniques in drug discovery, noting that gradient-based attribution, perturbation analysis, and attention-based visualization are now routinely applied to molecular property prediction, toxicity estimation, and *de novo* drug design. [75] provide an IEEE Access survey covering XAI across the pharmaceutical pipeline, emphasizing the growing regulatory imperative: both the FDA’s 2025 draft guidance and the EMA’s reflection paper require traceable, context-aware validation of AI systems in therapeutic development. siRNA models are directly affected because their predictions determine which candidates proceed to costly synthesis.

The XAI toolkit available to the biological sequence modeling community has matured considerably. At the foundation level, SHAP [76], grounded in cooperative game theory, provides a unified framework for decomposing any model’s prediction into additive feature contributions with formal guarantees of local accuracy, missingness, and consistency. SHAP has been widely adopted in genomics for identifying disease-relevant genes from expression data and in drug discovery for attributing molecular properties to specific chemical substructures. LIME [77] offers a complementary model-agnostic approach by fitting locally interpretable surrogates around individual predictions, enabling explanation of any black-box model regardless of architecture. For deep learning specifically, DeepLIFT [78] propagates activation differences through neural networks to assign contribution scores, and has become standard in regulatory genomics for identifying transcription factor binding motifs from DNA sequence models. [79] provide a cautionary complement, demonstrating that many saliency methods are unreliable under simple input transformations (such as constant shifts) that should not affect explanations, reinforcing the need for explicit faithfulness-validation protocols rather than visual inspection alone.

For graph neural networks, which are increasingly central to siRNA prediction (siRNADiscovery, ENsiRNA), GNNExplainer [80] generates explanations by identifying subgraph structures and node features most relevant to a given prediction through mutual information optimization. Adapting GNNExplainer to siRNA interaction graphs could reveal which base-pairing edges, structural contacts, or AGO2-interaction features drive specific knockdown predictions, providing mechanistic insight beyond what attention weights alone can offer.

Examples from neighboring biological domains illustrate interpretability advances directly applicable to siRNA prediction. In regulatory genomics, [81] developed Enformer, a transformer model for base-resolution gene expression prediction from DNA sequence. Enformer’s attention maps reveal long-range regulatory interactions spanning hundreds of kilobases, and its in-silico mutagenesis analyses identify specific sequence elements (enhancers, insulators, splice sites) that drive expression predictions. The interpretability methodology, generating variant effect predictions by computationally mutating individual positions and measuring prediction changes, is directly analogous to the saliency validation approach for siRNA. In cancer genomics, [68] constrained a neural network architecture to reflect known biological pathways, making internal representations correspond directly to molecular mechanisms (pathway-level activations) rather than opaque learned features. In gene regulation, [69] reviewed how attention, attribution, and in-silico mutagenesis extract regulatory grammar from chromatin accessibility models. In molecular property prediction, concept whitening aligns latent representations with known molecular descriptors [82]. [83] survey XAI in bioinformatics, categorizing methods into self-explainable (transparent by design) and supplemental (post-hoc), arguing that clinical applications must move toward the former. [84] introduce ShiftSmooth, a robust attribution mapping technique for transcription factor binding site prediction that accounts for sequence shifts, providing more reliable motif discovery than standard gradient methods.

These diverse examples share a common lesson: interpretability in biological AI is most valuable when it connects model internals to known biological mechanisms, enabling researchers to assess whether a model has learned genuine biology or exploited dataset artifacts. For siRNA prediction, this translates to verifying that attention patterns, saliency maps, and feature attributions correspond to established RNAi mechanisms (seed recognition, end asymmetry, target accessibility) rather than confounds introduced by experimental protocols.

Four concrete next steps would advance siRNA interpretability toward clinical-grade trustworthiness. First, *prospective experimental validation*: designing siRNAs where predictions hinge on specific saliency-highlighted positions, then testing mutations at those positions in the wet lab to verify that experimental results match predicted importance. This gold standard has not been achieved for any siRNA prediction model. Second, *biologically structured architectures*: following the Elmarakeby *et al*. paradigm, constraining model architecture to mirror RNAi pathway components (RISC loading module, seed recognition module, cleavage module) so that internal representations are inherently interpretable. Third, *counterfactual explanations*: generating minimal sequence edits that flip predictions from effective to ineffective (or vice versa), providing mechanistically informative explanations of what makes specific siRNAs work. Fourth, *SHAP-based decomposition* [76]: applying Shapley values to decompose knockdown predictions into contributions from individual nucleotide positions, thermodynamic parameters, and structural accessibility features, providing a unified attribution framework with formal axioms (additivity, symmetry, dummy) that gradient-based methods lack.

Table 4 summarizes the interpretability methods currently used across siRNA prediction models and their validation status.

**Table 4.** Interpretability methods used in siRNA prediction models

Model	XAI method	Key finding	Validation	Limitation
OligoFormer	Gradient saliency, attention maps	A/G at pos 1–2, U at 18–19; seed focus	Qualitative only	No faithfulness test
BERT-siRNA	Attention visualization	$5^{\prime }$ guide-end focus	Qualitative only	Cross-domain transfer unclear
AttSiOff	Multihead attention	Seed and off-target regions	FDA drug validation	No formal saliency test
siRNADiscovery	Feature importance (ablation)	Interaction node features critical	Leakage-aware splits	No position-level attribution
[29]	Perturbation-based counterfactual	Tests saliency faithfulness; reports cross-dataset collapse	Statistical acceptance test (ICLR MLGenX 2026)	No experimental (wet-lab) validation
[15]	LASSO coefficients	Asymmetric motifs, position weights	Inherently interpretable	Linear model only

## Off-target prediction

Off-target effects and chemical modifications remain the two most critical challenges for therapeutic siRNA design. [34] first identified off-target silencing as pervasive, using microarray profiling to show siRNAs produce widespread expression changes beyond intended targets. [35] subsequently established that seed-region complementarity (guide strand positions 2–8 matching $3^{\prime }$ UTR sequences) drives these effects, providing the mechanistic basis for computational off-target assessment. Chemical modifications, essential for therapeutic stability, compound the challenge by altering the molecular features that prediction models rely upon, creating tension between unmodified training data and the modified molecules deployed clinically.

### Empirical off-target assessment

#### SeedMatchR

SeedMatchR [85] provides a modern computational framework for empirical off-target assessment directly from RNA-seq data, replacing earlier microarray-based approaches with transcriptome-wide analysis. The tool takes differential expression results from siRNA transfection experiments and tests for enrichment of seed-matched sequences among downregulated transcripts using empirical cumulative distribution function (ECDF) analysis. By analyzing over 50 RNA-seq datasets, SeedMatchR establishes that 6-mer seed matches (positions 2–7 of the guide strand) are the most broadly predictive feature for off-target effects, though 7-mer and 8-mer matches produce stronger individual off-target signatures when they occur. The tool also quantifies how the abundance of seed-matched transcripts in the cell type of interest modulates off-target severity, providing cell-type-specific risk profiles that static sequence-based predictions cannot capture. SeedMatchR thus provides the transcriptome-wide auditing infrastructure that should become standard practice in any therapeutic siRNA design workflow.

#### Mechanistic seed region dissection

[71] provide the most detailed mechanistic dissection of seed-region function available, revealing through thermodynamic analysis combined with machine learning that the seed contains two functionally distinct domains. Positions 2–5 are essential specifically for off-target activity, acting as the primary determinant of unintended transcript binding. Positions 6–8 contribute to both productive RNAi silencing and off-target effects, meaning they cannot be modified to reduce off-target activity without also compromising on-target potency. Positions 8–14 show a negative correlation with off-target activity, suggesting that features in this region actively disfavor unintended interactions. This fine-grained mechanistic mapping provides both interpretable features for computational off-target models and explains the empirical observation that certain chemical modifications at specific seed positions can preferentially reduce off-target activity while preserving on-target potency.

#### Safe-seed design and experimental controls

[86] advanced a specificity-first design philosophy for Huntington’s disease therapy, prioritizing siRNA candidates whose seed sequences exhibit minimal complementarity to the expressed transcriptome of the target tissue. [87] generalized this into the siSPOTR tool, which scores seed off-target potential (POTS) transcriptome-wide for human and mouse. Incorporating such seed-safety screening at the design stage substantially reduces off-target signatures while maintaining potent on-target silencing. At the experimental validation level, C911 mismatch controls [88] provide a rigorous method for distinguishing seed-driven off-target effects from genuine on-target silencing. C911 controls preserve seed pairing with off-target transcripts while disrupting the central region required for on-target cleavage, and their application has revealed that many phenotypes initially attributed to target gene knockdown actually arise from off-target seed-mediated activity, emphasizing the essential role of computational off-target screening before synthesis and the danger of interpreting knockdown phenotypes without appropriate controls.

#### Web-based off-target tools

Several integrated web tools have been developed to make off-target assessment accessible to experimental researchers without computational expertise. [89] developed pssRNAit, a server for efficient off-target scoring of plant siRNAs that evaluates thermodynamic properties, target accessibility, and transcriptome-wide seed complementarity through an automated pipeline for agricultural RNAi applications. [90] contributed siRNA-Finder (si-Fi), a tool that integrates bowtie-based off-target mapping with RNAfold accessibility calculations, providing genome-wide off-target detection for user-supplied siRNA sequences with visualization of binding sites and thermodynamic profiles. These tools democratize off-target assessment by packaging complex computational analyses into accessible interfaces, though they rely on predefined algorithmic pipelines rather than the learned representations used by deep learning approaches.

### Structure-based chemical features

61] developed a systematic framework for generating reproducible structure-based features for off-target prediction. Working with over 30 000 siRNA/gene data points from RNA-seq experiments, they compared nine distinct feature representation strategies spanning sequence-only encodings, structural distance metrics, and molecular fingerprints. ECFPs computed from energy-minimized siRNA/AGO2 structural alignments achieved the highest predictive performance, demonstrating that incorporating the three-dimensional geometry of the protein/RNA interaction substantially improves off-target modeling beyond what sequence features alone can capture. The study provides an important lesson: feature representation choices (fingerprints versus structural distances versus sequence-only encodings) materially affect generalization, and the field should invest more systematically in comparing representation strategies rather than solely focusing on model architecture.

## Chemical modification

Chemical modifications are required for therapeutic siRNAs to achieve adequate nuclease resistance, reduced immunostimulation, and favorable pharmacokinetics, yet they alter the molecular features that efficacy models rely on, creating a tension between unmodified training data and the modified molecules deployed clinically. Computational prediction for modified siRNAs has a longer history than the recent deep-learning literature suggests, and we present it in roughly chronological order, from early sequence and cheminformatic models through random-forest methods to multiview deep learning and molecular-dynamics-based mechanistic analysis.

### Early sequence and cheminformatic models

The earliest dedicated effort, SMEpred [91], trained support vector machines on 3031 chemically modified siRNAs drawn from the siRNAmod database, using mononucleotide and dinucleotide composition and binary-pattern features to reach a Pearson correlation of 0.80, and was the first server to predict efficacy for modified rather than only unmodified siRNAs. Dong and Zheng [92] then introduced a cheminformatics representation, describing each nucleotide by 12 BCUT descriptors of charge, hydrophobicity, and polarity, giving 252 descriptors per 21-mer, and fitting partial-least-squares models; trained on the Huesken set for natural siRNAs and the smaller Bramsen modified-siRNA set [93], their models reached a predictive $r^2$ of 0.65, and were the first to characterize modified nucleotides by chemical structure rather than symbolic identity.

### Random forest models on the modified-siRNA benchmark

[94] addressed the small-data problem directly with an asymmetric trichotomous, two-threshold partitioning scheme that splits sequences into effective, undefined, and ineffective classes. On a dataset of 356 chemically modified siRNAs targeting seventeen genes, their random-forest model (benchmarked elsewhere as Monopoli-RF) outperformed a linear baseline and proved predictive in prospective experiments, with seven of ten top-ranked siRNAs achieving potent silencing. Building directly on this dataset, [37] provides a systematic three-algorithm comparison, evaluating Random Forest, SVM, and neural network classifiers across multiple efficacy thresholds on the same 356-sequence corpus. The study finds that Random Forest achieves the best AUC while SVM provides superior feature interpretability, revealing which modification patterns most strongly associate with efficacy retention or loss. Because both studies draw on the same corpus, they are best read together: [94] contributes the partitioning methodology, the random-forest result, and the dataset, while [37] contributes the comparison across algorithms and the interpretation of position-specific modification importances. Their shared reliance on a single small dataset underscores the data-scarcity barrier discussed in the ‘Future directions’ section.

### Cm-siRPred: multiview learning

Cm-siRPred [95] introduces multiview learning for chemically modified siRNA efficacy prediction. The model encodes three complementary views of each modified siRNA: double-strand nucleotide sequences, chemical modification patterns specifying the type and position of each modification ($2^{\prime }$-O-methyl, phosphorothioate, locked nucleic acid), and computed physicochemical properties. Cross-attention mechanisms enable the model to learn interactions between these views, capturing how specific modifications at specific positions modulate the sequence-determined baseline efficacy. Validation against five FDA-approved siRNA therapeutics and an accompanying webserver make Cm-siRPred among the most practically accessible tools for modification-aware prediction, directly addressing the gap between academic sequence-only prediction and the requirements of pharmaceutical development.

### Molecular dynamics and mechanistic insights

At the mechanistic level, [36] combined thermodynamic measurements with extensive molecular dynamics simulations to identify the structural determinants of modification tolerance. Their analysis reveals that low stabilization energies and increased sugar flexibility at guide-strand positions g2 and g6 correlate with higher activity across multiple modification chemistries including $2^{\prime }$-O-methyl, $2^{\prime }$-fluoro, GNA, and $5^{\prime }$-(E)-vinylphosphonate. These mechanistic insights serve dual purposes: they can directly inform feature engineering for modification-aware ML models, and they provide independent validation criteria for evaluating whether learned representations in models like ENsiRNA have captured genuine modification-activity relationships rather than dataset-specific correlations.

#### Chemically modified siRNA databases

Progress on modification-aware prediction depends on curated data, and two databases anchor the field. siRNAmod [62] was the first dedicated repository of experimentally validated chemically modified siRNAs, archiving 4894 entries spanning 128 unique chemical modifications with per-position annotation and, importantly for chemical-structure-aware modeling, storing each entry’s SMILES representation alongside efficacy, target gene, cell line, and assay metadata; it supplied the training data for SMEpred. CMsiRNAdb [96], released in 2026, substantially expands this resource by consolidating 43 153 experimentally validated sequences, of which 15 760 are unique, extracted from ninety patents and covering thirty-six modification types across thirteen therapeutic target genes. Beyond retrieval and visualization, CMsiRNAdb integrates ModMapper, a trie-based tool for precise identification of modification sites, and embeds the Cm-siRPred model for in-database efficacy evaluation. The patent-derived provenance of CMsiRNAdb is notable, since it captures the heavily optimized modification patterns used in actual therapeutic development that are otherwise absent from the academic literature, and it represents the most substantial public substrate yet available for training modification-aware models. Table 5 compares the two resources.

**Table 5.** Public databases of chemically modified siRNAs

Database	Year	Entries	Mod. types	Source	Key features
siRNAmod [62]	2016	4,894	128	Literature	SMILES annotation; per-position modifications; training data for SMEpred
CMsiRNAdb [96]	2026	43,153 (15,760 unique)	36	90 patents	ModMapper site identification; integrated Cm-siRPred; 13 target genes

### Comparative synthesis: Off-target tools, experimental datasets, and modification strategies

The preceding subsections treated off-target assessment and chemical modification through individual methods. Here we consolidate them, comparing off-target prediction strategies head to head, surveying the publicly available laboratory-derived datasets that underpin them, and answering directly which tools are driven by experimental data.

Table 6 compares the principal off-target prediction approaches and separates them along the axis the field most often conflates: whether a method is derived from experimental off-target measurements or instead scores candidate specificity from sequence and thermodynamic features. Two methods are experimental-data-driven. SeedMatchR [85] operates directly on RNA-seq differential-expression results, testing for enrichment of seed-matched transcripts among down-regulated genes, and is therefore empirical by construction. The structure-based framework of [61] goes further by training machine-learning models on >30 000 experimental siRNA/gene off-target data points, using ECFPs and energy-minimized siRNA/AGO2 structural alignments as features. The remaining tools, including si-Fi [90] and pssRNAit [89], enumerate seed-complementary sites and weight them by accessibility, and the off-target module of AttSiOff [24] counts transcriptome-wide seed matches alongside its efficacy prediction; these score specificity from sequence and structure and are validated against, but not trained on, experimental off-target data. The transcriptome-aware seed-safety scoring of [87] occupies an intermediate position, benchmarking sequence-derived safety scores against expressed-transcript repertoires.

**Table 6.** Comparison of siRNA off-target prediction approaches, distinguishing empirical RNA-seq-based methods from sequence/thermodynamic and structure-based predictors

Tool/method	Approach	Data basis	Exp.-data-driven?	Output/availability
SeedMatchR [85]	Seed-match enrichment among down-regulated transcripts (ECDF)	RNA-seq differential expression	Yes (empirical)	Per-transcript off-target enrichment; Bioconductor R package
Richter & Admasu [61]	Structure-based ML: ECFP fingerprints + siRNA/AGO2 structures (AutoML)	*> * 30 000 siRNA/gene RNA-seq points	Yes (trained on exp. data)	Off-target class probability; code and data with paper
siSPOTR [87]	Transcriptome-wide seed-safety (POTS) scoring	Expressed 3$^{\prime }$-UTR seed census	Benchmarked versus exp.	Seed-safety score; web tool
si-Fi [90]	Bowtie seed mapping + RNAfold accessibility	Sequence + thermodynamics	No (predictive)	Off-target hit list + accessibility; GitHub/webserver
pssRNAit [89]	Seed complementarity + accessibility (plant transcriptomes)	Sequence + thermodynamics	No (predictive)	Off-target hits; webserver
AttSiOff off-target module [24]	Transcriptome seed-match counting with efficacy	Sequence versus transcriptome	Partial	Off-target site count; GitHub/webserver

Only SeedMatchR and the structure-based framework of Richter & Admasu are derived directly from experimental off-target measurements; the remaining tools score candidate specificity from sequence and thermodynamic features and are validated against, rather than trained on, experimental off-target data.

Publicly available laboratory-derived off-target resources remain scattered rather than standardized. The most accessible include the rat-liver Ttr RNA-seq series distributed as example data with SeedMatchR, which was generated to demonstrate seed-region modification effects; the compendium of >30 000 siRNA/gene RNA-seq data points assembled by [61]; the >50 RNA-seq datasets analysed by [85]; and the classic microarray off-target profiles of [34, 35], which remain a reference point for seed-mediated silencing. Crucially, none of these constitutes a community-standardized off-target benchmark comparable to the Huesken efficacy dataset, a gap we return to in the ‘Future directions’ section.

Finally, Table 7 maps the chemical modifications that dominate therapeutic siRNA, namely 2$^{\prime }$-O-methyl, 2$^{\prime }$-fluoro, phosphorothioate, locked nucleic acid, GNA, and 5$^{\prime }$-(E)-vinylphosphonate, to the computational methods that model their effect on efficacy and specificity. Modification-aware prediction is currently led by Cm-siRPred [95], which encodes modification type and position as a distinct view, and ENsiRNA [27], which represents modifications as attributes on a geometric graph, while the thermodynamic and molecular-dynamics analysis of [36] supplies mechanistic determinants of modification tolerance that can be used as features or as independent validation criteria.

**Table 7.** Common therapeutic siRNA chemical modifications and the computational methods that model their effect on efficacy and specificity

Modification	Primary purpose	Computational modeling
2$^{\prime }$-O-methyl (2$^{\prime }$-OMe)	Nuclease resistance; seed-region off-target suppression; reduced immunostimulation	Cm-siRPred [95]; ENsiRNA [27]; [37]
2$^{\prime }$-fluoro (2$^{\prime }$-F)	Nuclease resistance; duplex stabilization	ENsiRNA [27]; [36]
Phosphorothioate (PS) backbone	Exonuclease resistance; improved pharmacokinetics	Cm-siRPred [95]
Locked nucleic acid (LNA)	Affinity and specificity tuning	Cm-siRPred [95]
GNA; 5$^{\prime }$-(E)-vinylphosphonate	Seed off-target mitigation; metabolic stability and 5$^{\prime }$-phosphate mimicry	[36] (thermodynamics + MD)

### Modification strategies and the off-target intersection

Off-target effects and chemical modification are distinct problems, the former concerning specificity and the latter stability, immunogenicity, and pharmacokinetics, but they intersect at a specific and consequential point: chemical modifications placed within the seed region can selectively reduce off-target activity. The mechanistic basis is the seed-domain dissection of [71], which shows that seed positions 2–8 govern off-target binding, and the position-specific findings of [36], which identify modifications at particular guide-strand positions that preserve on-target potency while altering off-target behavior. A 2-prime-O-methyl modification at a seed position, for example, can suppress seed-mediated off-target binding without abolishing on-target silencing. This intersection is where the off-target methods of the ‘Off-target prediction’ section and the modification-aware predictors of this section must ultimately be unified, and it motivates the joint efficacy-and-off-target, modification-aware models discussed in the ‘Future directions’ section. Table 7 summarizes the modification strategies and the computational methods that model them.

## Comparative analysis and method taxonomy

### Taxonomy of approaches

Table 8 provides a structured comparison of the major computational approaches for siRNA design, organized by method family.

**Table 8.** Taxonomy of computational approaches for siRNA design

Method family	Representative	Key features	Strengths	Limitations
Empirical rules	[8], [9]	Positional preferences, end asymmetry	Interpretable, fast, no training needed	Cannot capture interactions
Thermodynamic scoring	[11], [12]	*Δ G* , accessibility, composite scores	Physics-grounded, robust	Linear combinations only
Classical ML	[13], [15]	ANN, linear models on engineered features	Captures nonlinear interactions	Requires manual feature engineering
CNN-based	[20], [39]	Multikernel convolution, thermodynamic features	Local motif detection	Limited global context
Transformer	OligoFormer (2024), AttSiOff (2024), BERT-siRNA (2024)	Self-attention, foundation model embeddings, thermodynamics, interpretability, biology-informed learning	Long-range dependencies, transfer learning	Data-hungry, limited structural encoding
GNN	siRNADiscovery (2024), ENsiRNA (2025)	Interaction graphs, 3D structure, AGO2 features	Explicit topology encoding, leakage-aware	Smaller pretraining ecosystem
Foundation models	RNA-FM (2022), RiNALMo (2025), RNAGenesis (2024)	Pretrained embeddings from millions of RNAs	Transferable representations	Not siRNA-specific
Modification-aware	Cm-siRPred (2024), ENsiRNA (2025), [37]	Chemical modification encoding, multiview learning	Therapeutic relevance	Limited training data

Only the modification-aware family is designed for chemically modified siRNAs; all other families are developed and evaluated on unmodified data.

### Benchmark datasets

An important distinction underlies every dataset and performance figure in this section. The standard knockdown-efficacy benchmarks consist almost entirely of chemically unmodified siRNAs, carrying at most standard terminal overhangs, whereas the siRNAs used therapeutically are fully chemically modified. The three datasets in Table 9, Huesken, Takayuki, and Mixset, and therefore all of the head-to-head results in Table 10, are unmodified-siRNA datasets. Chemically modified efficacy data are far smaller and more heterogeneous, are treated separately in the ‘Chemical modification’ section, and are both more therapeutically relevant and more computationally challenging, because per-position chemistry, modified-duplex thermodynamics, and patterned modification schemes add degrees of freedom absent from the unmodified setting. We make this distinction explicit wherever the underlying data permit, and we caution that performance figures obtained on unmodified benchmarks should not be read as estimates of modified-siRNA performance.Reproducible evaluation requires shared benchmark datasets. Table 9 summarizes the three datasets most widely used for siRNA knockdown efficacy prediction. All three were aggregated and standardized by OligoFormer [23] and have since been adopted by DeepSilencer, siRNADiscovery, and other recent methods. Knockdown values are normalized to 0%–100% inhibition, with 70% as the positive/negative threshold. Sequences are truncated to 19 nucleotides for uniform processing.

**Table 9.** Benchmark datasets for siRNA knockdown efficacy prediction

Dataset	Year	siRNAs	mRNA targets	Assay type	Cell line	Key characteristic
Huesken	2005	2,431	34 human/rodent	Dual-reporter (eYFP/eCFP)	H1299	Largest single-lab dataset; BIOPREDsi training set
Takayuki	2007	583	4	Luciferase reporter	HeLa	Position-specific rules for every 3rd nucleotide
Mixset	Various	700	37	Mixed (qRT-PCR, reporter, Western)	Multiple	Merged from 7 studies; heterogeneous conditions

These three datasets, aggregated and standardized by [23], are used by the majority of recent methods for intra-dataset and inter-dataset evaluation. All three are chemically unmodified siRNA datasets; chemically modified efficacy datasets are described separately in the ‘Chemical modification’ section.

**Table 10.** Head-to-head performance comparison on standard benchmark datasets

Method	AUC (Huesken)	AUC (Mixset)	AUC (Takayuki)	PCC (Huesken)	Evaluation protocol
DSIR	0.85	0.78	0.72	—	Five-fold CV
i-Score	0.74	0.78	0.74	—	Five-fold CV
s-Biopredsi	0.78	0.80	0.76	—	Five-fold CV
OligoFormer	**0.862**	0.845	**0.863**	0.711	Five-fold CV
DeepSilencer	0.855	**0.858**	0.857	—	Five-fold CV
*Leakage-aware evaluation (siRNA-split on Huesken)*					
siRNADiscovery	0.874	—	—	**0.770**	siRNA-split, 10 seeds
GNN4siRNA	0.821	—	—	0.679	siRNA-split, 10 seeds

Top section: five-fold cross-validation results reported by [23] and [63]. Bottom section: leakage-aware siRNA-split results from [26]. Bold indicates best within each evaluation protocol. AUC values are approximate where extracted from figures. PCC = Pearson correlation coefficient. All results are on chemically unmodified benchmarks and should not be interpreted as estimates of modified-siRNA performance.

### Head-to-head performance comparison

Table 10 compiles reported performance metrics from methods evaluated on the same benchmark datasets under comparable conditions. Values for OligoFormer, DSIR, i-Score, and s-Biopredsi are from five-fold cross-validation reported by [23]. siRNADiscovery results are from siRNA-split evaluation on the Huesken dataset reported by [26]. DeepSilencer results are from [63]. Because evaluation protocols differ across studies (five-fold CV versus siRNA-split versus mRNA-split), direct comparisons across rows should be made cautiously; the leakage-aware protocols of [26] produce systematically lower numbers than standard five-fold CV. This heterogeneity underscores the urgent need for community-standardized benchmarks.

### Code and tool availability

Reproducibility depends on open access to implementations. Table 11 summarizes the availability of code, pretrained models, and webservers for the methods reviewed. Open-source availability varies considerably: recent deep learning methods generally provide GitHub repositories, while classical methods are primarily available as webservers.

**Table 11.** Code and tool availability for siRNA prediction methods reviewed in this paper

Method	Year	Code Repository	Webserver	Notes
DSIR	2006	—	*\checkmark*	Web tool; linear model
i-Score	2007	—	*\checkmark*	Web tool; composite scoring
OligoFormer	2024	github.com/lulab/OligoFormer	—	Docker image available
AttSiOff	2024	github.com/Bioxai/AttSiOff	*\checkmark*	End-to-end pipeline
BERT-siRNA	2024	github.com/nxu1/BERT-siRNA	—	Pretrained weights included
DeepSilencer	2025	github.com/BlackCattt9/DeepSilencer	—	Data from OligoFormer
siRNADiscovery	2024	github.com/lzr-r/siRNADiscovery	—	Leakage-aware splits provided
ENsiRNA	2025	github.com/Amadeoy/ENsiRNA	—	3D + modification support
Cm-siRPred	2024	—	*\checkmark*	Modified siRNA webserver
SeedMatchR	2024	Bioconductor R package	—	Off-target RNA-seq analysis
si-Fi	2019	github.com/snowformatics/siRNA	*\checkmark*	Off-target with RNAfold
pssRNAit	2020	—	*\checkmark*	Plant siRNA off-target

Webserver availability indicated by *\checkmark*. Repository URLs are abbreviated for readability.

### Feature landscape and nonredundancy

**Figure 4.**
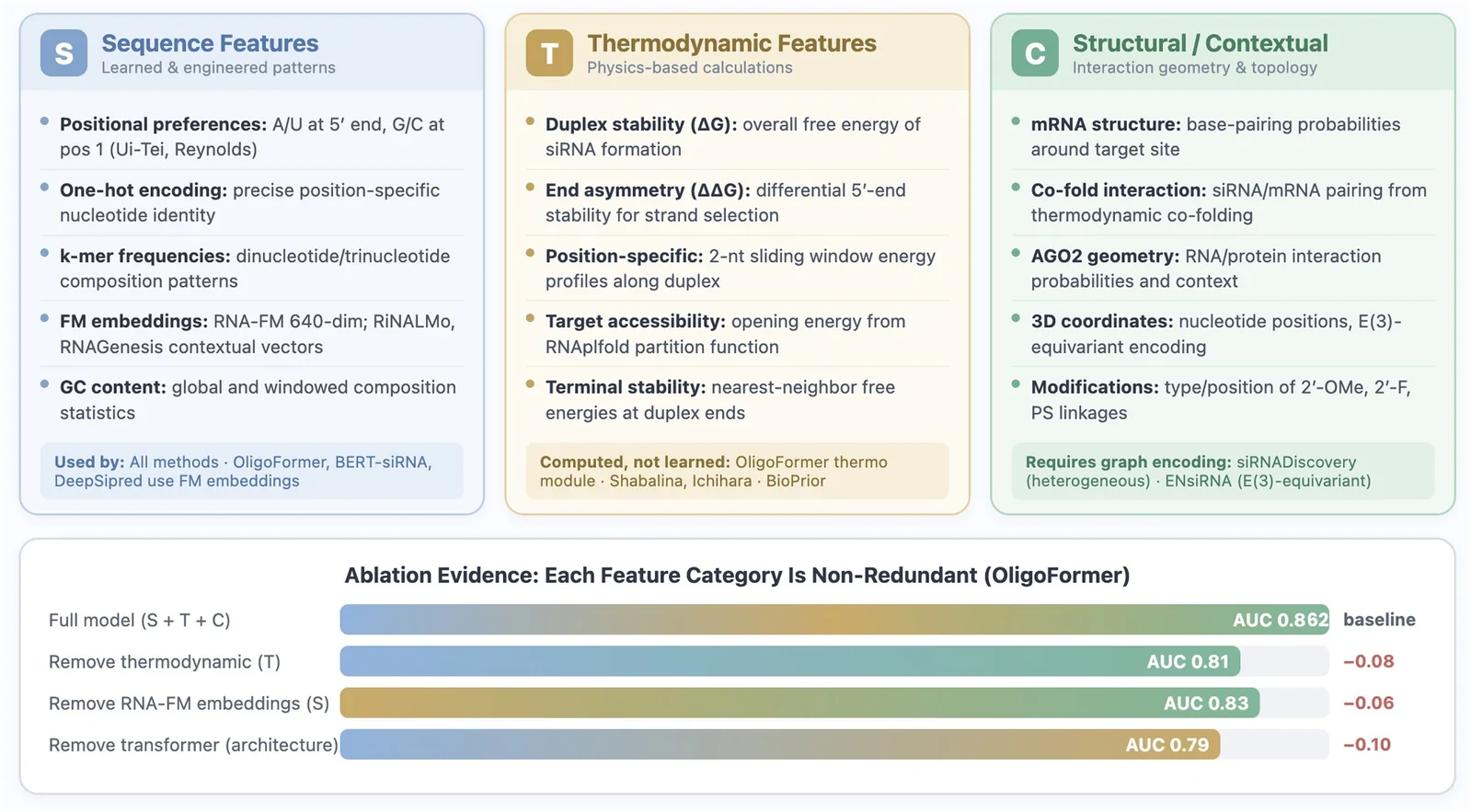
Feature taxonomy for siRNA knockdown efficacy prediction. Top: three complementary feature categories (sequence, thermodynamic, structural/contextual) with representative features, source models, and computation methods. Bottom: OligoFormer ablation evidence confirming nonredundancy across categories; removing any single feature type degrades AUC, with the transformer architecture contributing the largest individual effect.

A consistent finding is that predictive features for siRNA knockdown efficacy fall into three broadly complementary categories (Fig. 4), and the best-performing models leverage all three rather than relying on any single category.

*Sequence features* capture positional nucleotide preferences and compositional statistics that reflect the biochemistry of RISC loading and target engagement. These can be represented via one-hot encoding (preserving precise positional information), k-mer frequencies (capturing local motif patterns at the cost of positional resolution), or pretrained foundation model embeddings (encoding contextual patterns learned from millions of RNA sequences). The choice of representation matters: siRNADiscovery demonstrated that one-hot encoding consistently outperforms k-mers for siRNA prediction because position-specific nucleotide identity is mechanistically more relevant than local composition patterns. Foundation model embeddings subsume both representations by encoding both positional and contextual information in high-dimensional learned spaces.

*Thermodynamic features* encode the physics of RNA hybridization and structure formation. These include overall duplex stability (*Δ G*), end asymmetry ($\Delta \Delta G$) that governs strand selection, position-specific stabilities computed across sliding windows, and target-site accessibility estimated from partition-function methods [32]. Crucially, these features are computed from established biophysical models rather than learned from siRNA efficacy data, meaning they provide an independent source of information that does not suffer from the same overfitting risks as learned features. Thermodynamic features remain indispensable: OligoFormer’s ablation studies confirm that removing thermodynamic features degrades AUC even with powerful foundation model embeddings (quantified in Table 3), showing that current deep learning models have not learned thermodynamic principles from data alone.

*Structural and contextual features* capture the three-dimensional and interaction context that determines whether an siRNA can productively engage its target within the cellular environment. These include mRNA secondary structure around the binding site, siRNA/mRNA base-pairing probabilities from thermodynamic co-fold analysis, and the geometry of the AGO2/siRNA/mRNA ternary complex. GNN architectures (siRNADiscovery, ENsiRNA) encode these naturally as graph topology; transformer architectures incorporate them as concatenated input features. ENsiRNA’s use of explicit 3D nucleotide coordinates through E(3)-equivariant networks represents the most direct structural encoding to date.

The nonredundancy of these feature categories is established through ablation studies in multiple architectures. OligoFormer, AttSiOff, siRNADiscovery, and DeepSipred each report that removing any single feature category degrades performance, confirming that sequence patterns, thermodynamic properties, and structural context carry genuinely complementary information about knockdown efficacy. The practical implication is clear: future architectures should better integrate these categories rather than replacing one with another.

### Evaluation standards and the leakage problem

The most consequential recent finding is that evaluation protocols profoundly affect reported performance and can systematically mislead method comparisons. [26] demonstrated through careful analysis that standard random cross-validation for siRNA prediction allows multiple forms of data leakage: siRNAs targeting the same mRNA transcript can appear in both training and test partitions, and in some cases identical or near-identical siRNA sequences measured under different experimental conditions appear on both sides of the split. Because models can partially memorize target-specific or sequence-specific patterns from training, this leakage inflates test-set performance in ways that do not reflect genuine generalization to novel siRNAs or novel targets.

Under the leakage-aware splitting protocols proposed by Long *et al*. (siRNA-split and mRNA-split), several important findings emerge. First, the absolute performance of all methods decreases, often substantially. Second, the performance gaps between methods narrow, suggesting that some previously reported advantages were artifacts of leakage rather than genuine architectural superiority. Third, methods with stronger inductive biases (GNNs with explicit interaction encoding) tend to degrade less under strict splitting than methods with weaker inductive biases (simple CNNs), suggesting that structural encoding helps genuine generalization.

Complementary benchmark resources have emerged to support rigorous evaluation. RNATx-Bench [44] provides a multitask benchmark spanning six RNA modalities including siRNA efficacy. siRNAEfficacyDB [97] consolidates published experimental datasets with standardized annotations. These resources, combined with the Long *et al*. splitting protocols, provide the infrastructure for fair and reproducible comparisons. However, the community has not yet converged on a single accepted benchmark protocol, and papers continue to be published using evaluation setups that are vulnerable to the leakage problems identified by Long *et al*. Establishing community consensus on evaluation standards, analogous to the CASP assessments in protein structure prediction, should be a high priority.

## Future directions

**Figure 5.**
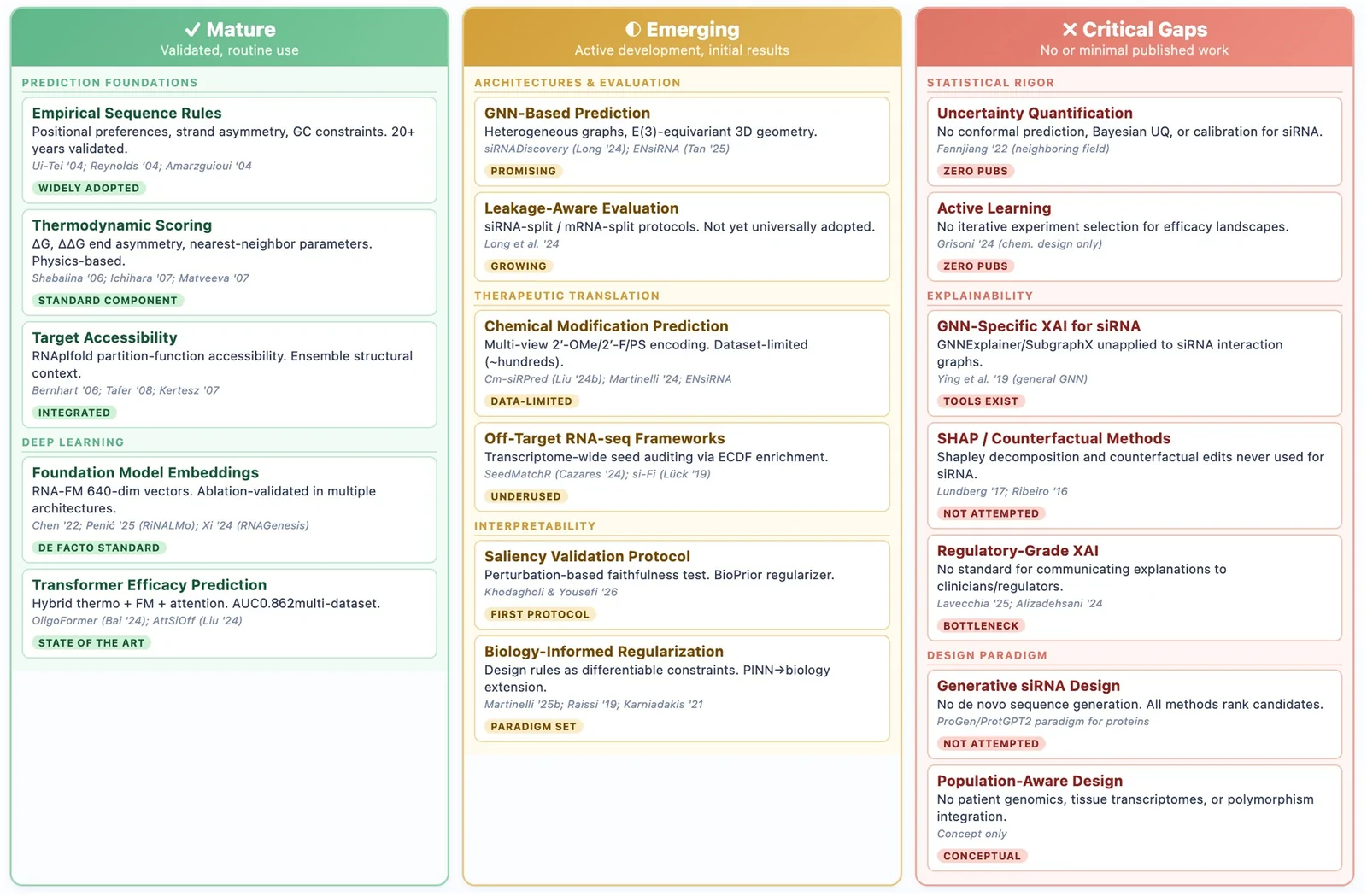
Research maturity landscape for computational siRNA design (2025). Mature methods (left, green) are validated and in routine use. Emerging methods (center, amber) show initial results with active development. Critical gaps (right, red) identify high-impact opportunities with no or minimal published work, organized by statistical rigor, explainability, and design paradigm.

The rapid progress documented in this review has resolved many early challenges in computational siRNA design, but has simultaneously exposed deeper problems and new opportunities (Fig. 5 summarizes the current research maturity landscape). In this section we identify the most promising research directions, distinguishing between near-term opportunities where existing tools could be immediately applied and longer-term visions that require fundamental methodological advances.

### Uncertainty quantification: the most critical gap

The most striking omission in siRNA prediction is formal uncertainty quantification (UQ). Despite siRNA design being safety-critical, no published method provides calibrated confidence intervals alongside point estimates. This stands in sharp contrast to neighboring fields: conformal prediction has been applied to drug-activity prediction [98], Bayesian deep learning is routine in molecular property prediction, and evidential deep learning has been explored for protein function annotation.

The opportunity is concrete and immediate. Conformal prediction methods provide distribution-free coverage guarantees and can be applied as a post-hoc wrapper around any existing predictor, requiring only a held-out calibration set. For siRNA prediction, conformal sets would provide guaranteed coverage (e.g. ‘the true knockdown lies in this interval with 90% probability’) that accounts for differences between training and deployment distributions across cell types, assay protocols, and modification chemistries. Bayesian approaches could provide posterior uncertainty that guides experimental prioritization, directing synthesis resources toward candidates where the model is confident rather than uncertain. Temperature scaling, the simplest calibration technique, could improve the reliability of existing model outputs with minimal computational overhead. The observation discussed in the ‘Biology-informed learning and interpretability’ section, that explanation faithfulness can collapse when the assay context shifts, makes UQ especially urgent: uncertainty estimates could flag deployment contexts where predictions are unreliable before experimental resources are committed.

### Active learning for experimental design

Active learning, iteratively selecting maximally informative experiments, has no dedicated siRNA publications despite clear applicability. The standard predict-synthesize-test workflow is inherently sequential and could be formalized as an active learning loop that selects candidates maximizing information gain rather than predicted efficacy, revealing which features the model understands poorly.

Methods combining reinforcement learning with active experimental design from chemical science [99] could dramatically reduce the number of siRNAs that must be synthesized and tested to achieve a desired prediction accuracy. Bayesian optimization over sequence space, guided by Gaussian process surrogates or neural network uncertainty estimates, offers another natural formulation. The practical barrier is the turnaround time of siRNA experiments (typically days to weeks from synthesis to readout), but computational pre-screening could identify batches of maximally informative candidates for parallel synthesis.

### Population-aware personalized design

Current models predict average knockdown efficacy across cell lines, but therapeutic siRNAs must work in specific patient populations with distinct genetic backgrounds and disease states. A population-aware design system could combine OligoFormer-style efficacy prediction with transcriptome census data, computing seed and supplementary pairing landscapes against the actually expressed $3^{\prime }$-UTR repertoire for specific tissues and disease contexts. Integration with large-scale genomic databases (gnomAD, UK Biobank) would enable designs that avoid common polymorphisms that could create patient-specific off-target binding sites or disrupt on-target efficacy in specific populations. SeedMatchR provides the transcriptome-wide auditing infrastructure necessary for cell-type-specific off-target risk assessment, and the combination of personalized efficacy prediction with personalized safety assessment is a natural endpoint for the field.

### Modification-aware hybrid architectures

The most practically impactful near-term architectural advance would be a unified model that handles both unmodified and chemically modified siRNAs within a single framework, jointly predicting efficacy, off-target risk, and metabolic stability. ENsiRNA shows that E(3)-equivariant 3D features improve prediction; Cm-siRPred shows that multiview modification encoding enables modified siRNA prediction; OligoFormer shows that transformer attention effectively integrates thermodynamic and foundation model features. A hybrid graph/transformer architecture could encode guide and target as attributed graphs with chemical modification tags at each node, base-pairing and stacking priors as edges, and an AGO2-context subgraph providing protein/RNA interaction geometry. Dual prediction heads for efficacy and off-target propensity would enable joint optimization across the two most critical therapeutic objectives. The principal obstacle is training data: curated datasets of chemically modified siRNAs with quantitative efficacy measurements remain small (hundreds of examples versus thousands for unmodified siRNAs), though pharmaceutical development programs are generating increasingly large proprietary datasets.

### Expanding and standardizing experimental data: modified-siRNA and off-target benchmarks

The architectural directions above are ultimately gated by data. The single most consequential barrier to clinical translation is not model capacity but the small, heterogeneous, and largely private experimental record on which siRNA models are trained and evaluated. Three needs are acute.

First, the field needs large-scale, publicly available chemically modified siRNA efficacy datasets. Therapeutic siRNAs are universally modified, yet the curated modified-siRNA corpora used by current models number in the hundreds, for example the 356-sequence set analysed by [37], against thousands of unmodified siRNAs available for efficacy prediction. The data needed to train modification-aware models exist, but largely as proprietary pharmaceutical datasets that are not shared. A public benchmark pairing sequence, the full modification pattern, and quantitative knockdown would be the highest-value data investment for the field and is a prerequisite for the modification-aware hybrid architectures described in the ‘Future directions’ section.

Second, the field needs standardized, laboratory-sourced off-target RNA-seq benchmarks. As detailed in the ‘Off-target prediction’ section, off-target evaluation currently relies on scattered RNA-seq studies and aggregated compendia with no community-standardized benchmark analogous to the Huesken efficacy dataset. A curated, transcriptome-wide off-target benchmark with consistent cell types, doses, chemistries, and read-out would allow empirical and structure-based off-target predictors to be compared on equal footing, much as the leakage-aware splits of [26] standardized efficacy evaluation.

Third, the field must explicitly mitigate inter-lab batch effects and improve data sharing. Because efficacy and off-target measurements vary with cell line, transfection protocol, dose, and assay, aggregating datasets across laboratories introduces batch effects that must be modelled rather than ignored. The finding by [29] that models trained on one assay type can transfer faithfully among similar assays yet collapse on a luciferase reporter shows that assay-specific confounds can silently invalidate both predictions and their explanations, and the leakage documented by [26] is a related symptom of insufficiently controlled data composition. Practical remedies include explicit batch correction with assay context as a covariate, FAIR deposition with standardized metadata covering cell line, dose, time point, chemistry, and platform, dataset-aware normalization of the kind already used by OligoFormer, and federated learning to train across proprietary datasets without exchanging raw data. Without these measures, leakage-aware splitting addresses only part of the reliability problem, since it controls how existing data are partitioned but not the heterogeneity within them.

### Physics-informed and biology-informed regularization

The broader physics-informed machine learning paradigm [66, 67] offers concrete benefits for siRNA prediction. Differentiable accessibility regularization, adding penalty terms during training proportional to the local structural opening cost around the target site from RNAplfold computations, provides a principled way to inject physical knowledge without sacrificing model flexibility. Biology-informed regularizers have been reported to improve not only prediction accuracy but the faithfulness of learned explanations (see the ‘Biology-informed learning and interpretability’ section), producing models whose explanations are more reliably aligned with known biology. [30] argues more broadly that biology-informed approaches connecting design principles as soft constraints improve both accuracy and mechanistic interpretability. Extending this paradigm to encode additional physical priors, such as strand-selection thermodynamics, RISC-loading kinetics, and nuclease degradation rates, could produce models that are not only more accurate but also more physically plausible and more trustworthy for clinical deployment.

### Toward explainable RNA therapeutics

The convergence of regulatory pressure, clinical stakes, and community maturation makes explainable AI for RNA therapeutics an inevitable and high-impact research direction. Current siRNA prediction models employ only the simplest XAI techniques (gradient saliency, attention visualization), but the broader XAI ecosystem offers substantially more powerful tools that remain unapplied.

Three specific opportunities stand out. First, *GNN-specific explainability* for graph-based siRNA models (siRNADiscovery, ENsiRNA) is entirely absent. GNNExplainer [80] and its successors (PGExplainer, SubgraphX) can identify which subgraph structures, edges, and node features drive individual predictions, potentially revealing which base-pairing interactions, structural contacts, or AGO2 features are most critical for specific siRNAs. Applying these methods to siRNA interaction graphs would yield interpretability at the interaction level rather than the position level, complementing the sequence-level saliency maps from transformer models.

Second, *multi-omics integration for context-aware explanations*. Current siRNA predictions are context-free: the same model produces the same prediction regardless of cell type, tissue, or disease state. Yet siRNA efficacy varies substantially across biological contexts due to differences in target expression levels, mRNA isoform usage, competing endogenous RNA networks, and intracellular delivery efficiency. Integrating transcriptomic data with siRNA prediction, then using XAI to explain how cellular context modulates predicted efficacy, would connect computational predictions to the biological reality of therapeutic deployment. SHAP decomposition could quantify how much of a prediction derives from intrinsic siRNA properties versus target-context features, guiding context-aware design.

Third, *regulatory-grade explanation standards*. As siRNA therapeutics move toward broader clinical deployment and regulatory agencies mandate AI transparency, the field needs standardized formats for communicating model explanations to noncomputational stakeholders (clinicians, regulators, patent examiners). This requires translating technical XAI outputs (attribution vectors, attention matrices, counterfactual perturbations) into domain-specific explanation languages that connect to established pharmacological concepts (potency, selectivity, therapeutic index). [74] and [75] both identify this translational gap as a critical bottleneck for XAI adoption in drug development.

### Cross-modality foundation models and scaling

The RNA foundation model ecosystem is expanding rapidly, and several scaling trajectories point toward models with direct relevance to siRNA design. The progression from RNA-FM (100M parameters) through RiNALMo (650M) to AIDO.RNA and RNAGenesis (1B+) suggests that larger models trained on more diverse corpora will continue to improve the quality of pretrained representations available for siRNA prediction. A critical near-term experiment, as noted in the ‘RNA foundation models as pretrained representations’ section, is the systematic evaluation of newer foundation models (RiNALMo, RNAGenesis, AIDO.RNA) as drop-in replacements for RNA-FM embeddings in existing siRNA prediction architectures.

Longer-term, cross-biopolymer foundation models that jointly tokenize nucleotide and amino acid sequences could enable direct reasoning about guide-strand/AGO2 interactions, mRNA target accessibility, and protein-context effects within a single learned representation. RNAGenesis’s strong cross-modality performance [44] and the industrial development of specialized models like REPRESS for miRNA–mRNA regulation [100], preprint) suggest that such cross-modality transfer is already beneficial. The Evo model [48], trained at the DNA+RNA level with 7 billion parameters, shows that genome-scale language models capture regulatory relationships that may include RNAi-relevant patterns.

## Conclusions

This review traced the computational siRNA design field from empirical sequence rules through thermodynamic scoring, classical learning, and current deep learning architectures integrating foundation models, attention mechanisms, graph reasoning, and biology-informed constraints. The arc from Fire *et al*.’s (1998) RNAi discovery to the hybrid models of 2024–2025 is a remarkably complete trajectory in computational biology. Several cross-cutting themes emerge.

**Hybrid approaches tend to outperform pure approaches, with important caveats**. The most effective knockdown prediction methods combine learned representations with physics-informed features rather than relying on either alone. OligoFormer’s integration of thermodynamic calculations, RNA-FM embeddings, and transformer attention exemplifies this pattern, with ablation studies showing that each component carries nonredundant information. Two caveats are important, however. First, this clearest quantitative evidence comes from a single model under a single, leakage-vulnerable five-fold protocol, and whether the per-module contributions persist under the leakage-aware evaluation of [26] is untested. Second, the claim should be stated as the value of integrating physical priors with learned representations, not as the necessity of foundation-model embeddings: DeepSilencer matches or exceeds OligoFormer on Mixset using only sequence and physicochemical features, with no foundation-model embeddings, so embeddings are evidently sufficient but not necessary for state-of-the-art performance. This finding is remarkably robust across architectures: siRNADiscovery’s combination of co-fold interaction features with learned graph representations, ENsiRNA’s fusion of 3D geometry with language model embeddings, and even classical methods like Shabalina *et al*.’s (2006) combination of thermodynamic and sequence features all reflect the same principle. Pure end-to-end learning without physics-based features tends to underperform approaches that integrate them in the siRNA domain, likely because the training datasets are too small and heterogeneous to learn thermodynamic principles from data alone.

**Foundation models address the fundamental data-scarcity problem**. The chronic shortage of experimentally validated siRNA knockdown data, typically thousands to low tens of thousands of labeled examples, has historically limited the capacity of supervised learning methods. Pretrained RNA language models circumvent this bottleneck by providing rich, transferable representations learned from millions of unlabeled RNA sequences. The scaling trajectory from RNA-FM (100 million parameters) through RiNALMo (650 million) to RNAGenesis (1 billion+) suggests that continued scaling will yield further improvements, though the practical question of which foundation model best serves siRNA prediction specifically remains to be answered through systematic head-to-head comparisons.

**Rigorous evaluation is as important as architectural innovation**. The demonstration by [26] that data leakage has systematically inflated benchmark results across the siRNA prediction field is a sobering finding that retroactively undermines many published performance comparisons. Their leakage-aware splitting protocols (siRNA-split and mRNA-split) should become minimum requirements for any future benchmark study. More broadly, the field needs standardized evaluation infrastructure comparable to what protein structure prediction has in CASP: common datasets, agreed-upon metrics, held-out test sets that are truly independent, and prospective experimental validation of computational predictions.

**Off-target assessment and modification awareness are maturing but incomplete**. The computational tools for transcriptome-wide off-target auditing (SeedMatchR, pssRNAit, si-Fi) and the mechanistic understanding of seed-region function [71] have advanced substantially. Modification-aware prediction (ENsiRNA, Cm-siRPred) is becoming tractable. However, these capabilities remain largely siloed: no existing system jointly optimizes sequence efficacy, off-target safety, and modification compatibility within a single end-to-end framework. Building such integrated systems is essential for therapeutic translation.

**Interpretability requires validation, not just visualization**. The standard practice of displaying attention maps or saliency heatmaps as evidence that models have learned biological mechanisms is insufficient. The argument developed in the ‘Biology-informed learning and interpretability’ section, that saliency faithfulness can fail silently and that explanations can collapse across assay contexts, indicates that interpretability claims require explicit statistical validation rather than visualization alone. Biology-informed regularizers that improve saliency faithfulness represent a practical mechanism for producing models whose explanations are trustworthy enough to guide experimental decisions.

**Critical gaps represent concrete opportunities**. UQ, active learning, population-aware personalized design, and hybrid graph/transformer architectures all represent well-defined research directions where existing methods from neighboring fields could be immediately applied to siRNA prediction. The absence of formal UQ in a safety-critical therapeutic domain is particularly notable and represents perhaps the highest-priority gap for the field to address.

The computational siRNA design field has matured to the point where modern prediction pipelines, integrating classical rules, thermodynamic calculations, foundation model embeddings, attention-based efficacy prediction, transcriptome-wide seed auditing, and modification optimization, represent a practical and increasingly indispensable component of therapeutic RNA development. The challenge ahead is not whether computational siRNA design works but how reliably and safely it can be deployed. Progress now depends as much on evaluation rigor, UQ, and experimental integration as on architectural novelty. The tools exist; the remaining task is to make the design process trustworthy enough for routine clinical use.

## Key points

- Integrating thermodynamic features with learned representations characterizes the strongest siRNA efficacy predictors, but competitive performance is achievable without foundation-model embeddings, so the evidence supports physical-prior integration more than foundation-model necessity.
- Data leakage in previous benchmarks has inflated reported performance; leakage-aware splitting protocols are essential for reliable evaluation.
- Graph neural networks and transformers offer complementary inductive biases for siRNA design; hybrid architectures remain underexplored.
- UQ and active learning represent significant untapped opportunities for therapeutic siRNA development.
- Chemical modification-aware prediction is emerging but requires larger curated datasets for therapeutic-grade performance.

## Data Availability

No new data were generated or analysed in support of this review article.
